# Comparative analysis reveals molecular adaptation of mammalian HCA_2_ to microbial metabolites

**DOI:** 10.1016/j.isci.2026.116030

**Published:** 2026-05-22

**Authors:** Franziska Bischof, Petra Krumbholz, Gunnar Kleinau, Patrick Scheerer, Claudia Stäubert

**Affiliations:** 1 Rudolf Schönheimer Institute of Biochemistry, Faculty of Medicine, Leipzig University, Leipzig, Germany; 2 Institute of Medical Physics and Biophysics, Group Structural Biology of Cellular Signaling, Charité – Universitätsmedizin Berlin, Corporate Member of Freie Universität Berlin, Humboldt-Universität zu Berlin, 10117 Berlin, Germany

**Keywords:** pharmacology, molecular biology, evolutionary biology, cell biology

## Abstract

Metabolite-sensing G protein-coupled receptors (GPCRs), such as hydroxycarboxylic acid receptor 2 (HCA_2_), translate endogenous and microbial signals into physiological responses, regulating metabolism and immunity, yet the extent of HCA_2_ functional diversification across mammals remain unclear. Here, comparative pharmacology, metabolomics, evolutionary analysis, and structural mapping of mammalian HCA_2_ orthologs reveal extensive functional diversification across mammals, especially in odd-toed ungulates. Notably, African rhinoceros HCA_2_ exhibits multiple HCA_3_-like substitutions, resulting in loss of responsiveness to HCA_2_ agonists and increased sensitivity to HCA_3_-specific ligands. Positive selection analyses and metabolomic profiling of fecal extracts implicate microbiome-derived metabolites, particularly phenylpropionic and *trans*-cinnamic acid, as potential drivers of this adaptive shift. Mutagenesis experiments identified key amino acid substitutions in extracellular and transmembrane regions that modulate ligand potency and efficacy. These findings demonstrate that mammalian HCA_2_ receptors have undergone lineage-specific molecular evolution shaped by host-microbe metabolic interactions, highlighting how ecological contexts drive receptor adaptation and functional diversification.

## Highlights

• Cross-species comparison reveals diversification of the metabolite receptor HCA_2_
• Evolutionary sequence variation reshapes HCA_2_ ligand recognition across mammals
• Rhinoceros HCA_2_ acquires HCA_3_-like pharmacology through defined substitutions
• Host metabolic and microbiome environments shape GPCR evolution

## Graphical abstract

**Figure:**
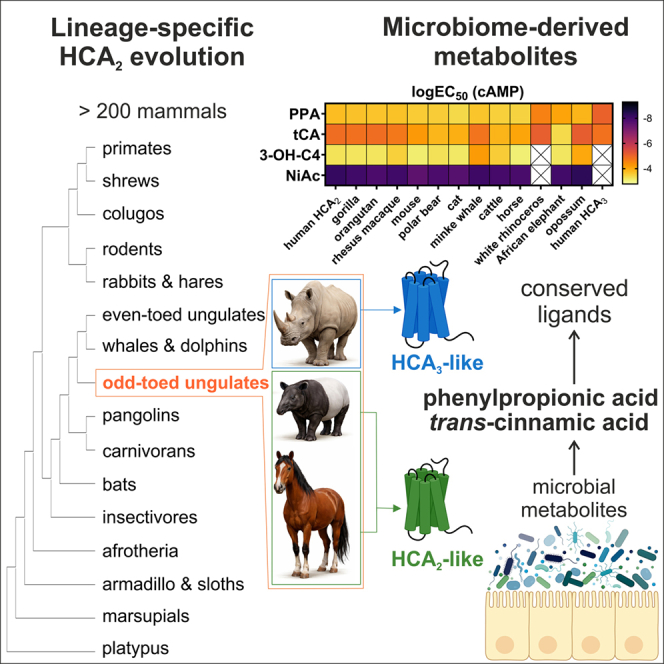

## Introduction

G protein-coupled receptors (GPCRs) play a central role in cellular communication, enabling organisms to detect and respond to a wide range of environmental, metabolic, and microbial signals.^1^ Their ability to recognize diverse ligands and engage multiple signaling pathways forms the basis of both their physiological versatility and pharmacological importance.^2^^,^^3^ Subtle sequence variations can profoundly influence ligand binding and receptor activation, driving the diversification of receptor function across species to fine-tune physiological responses to their ecological niches.^4^ Among these, metabolite-sensing GPCRs play a crucial role in sensing a remarkable array of metabolites originating from both host cells and the surrounding microbial environment, coordinating key physiological processes at the interface of metabolism, immunity, and health.^5^^,^^6^ However, the molecular basis of their evolutionary adaptation remains poorly understood.^7^ In particular, it remains unclear how evolutionary sequence variation in metabolite-sensing GPCRs reshapes ligand recognition while preserving conserved signaling architecture across species.

Hydroxycarboxylic acid receptors (HCAs) are prototypical metabolite sensors activated by endogenous and microbial metabolites. HCA_2_ (GPR109A) and its paralog HCA_3_ (GPR109B) modulate lipid metabolism, inflammatory processes, and barrier function through G_i/o_ protein-mediated signaling.^8^^,^^9^^,^^10^^,^^11^^,^^12^^,^^13^^,^^14^^,^^15^ In humans, HCA_3_ recognizes structurally related, lactic acid bacteria-derived aromatic acids, reflecting potential divergence from an ancestral metabolite-sensing function.^16^ This paralog divergence suggests that ligand-binding specificity within the HCA family is evolutionarily plastic, yet the molecular determinants of such diversification across mammals remain unresolved.

All these HCA_2_-mediated effects make the receptor an attractive target for anti-inflammatory and immunomodulatory drugs and highlight its critical role in protecting the host against pro-inflammatory insults. Especially the skin and gastrointestinal mucosa, where HCA_2_ is highly expressed, act as key defense barriers preventing microbial invasion and inflammation.^17^^,^^18^ The close association between HCA_2_ signaling and host defense suggests that the receptor operates at the interface of immunity and metabolism, in systems closely associated with the resident microbiota. Symbiotic relationships between commensal microorganisms and mammalian hosts are thought to influence the evolution of metabolite-sensing receptors.^19^^,^^20^

HCA_2_ was identified in 2003 as the molecular target of nicotinic acid (NiAc), a pharmacological ligand used clinically to treat dyslipidemia.^21^^,^^22^^,^^23^ Although NiAc activates HCA_2_ at supraphysiological concentrations, endogenous activation of the receptor predominantly arises from metabolic and microbial intermediates. While the therapeutic lipid-lowering effects of NiAc occur independently of HCA_2_, the clinically limiting side effect of skin flushing results from pharmacological HCA_2_ activation in epidermal Langerhans cells and keratinocytes via prostanoid release.^24^^,^^25^^,^^26^^,^^27^^,^^28^ Beyond pharmacology, NiAc and its vitamin equivalent nicotinamide (Nam) are also produced by gut microbes, linking HCA_2_ activity to host-microbiome metabolism.^6^^,^^29^^,^^30^

Physiologically, HCA_2_ is primarily activated by endogenous metabolites such as the ketone body 3-hydroxybutyrate (3-OH-C4), produced during fasting, and the microbial short-chain fatty acid butyrate (C4), positioning the receptor as a sensor of metabolic and microbiota-derived states.^31^^,^^32^

In humans and other mammals, several synthetic HCA_2_ ligands, including monomethylfumarate (MMF), acipimox, acifran, MK-6892, GSK256073, MK-0354, and LUF6283, have been developed over the past two decades.^33^^,^^34^^,^^35^^,^^36^^,^^37^^,^^38^ Many of these compounds show species-dependent potency differences, with EC_50_ values for MK-6892 varying more than 100-fold between human and rodent HCA_2_.^35^ Such disparities highlight the importance of considering evolutionary variation when translating pharmacological data across species, particularly in the context of drug development and comparative physiology.^39^^,^^40^ Given its dual role in metabolic sensing and immune regulation, HCA_2_ continues to be investigated as a therapeutic target for inflammatory and metabolic disorders, including psoriasis, Parkinson’s disease, and multiple sclerosis.^12^^,^^15^^,^^41^^,^^42^

Recent advances in GPCR structural biology have produced numerous high-resolution 3D structures of HCA_2_ bound to diverse ligands.^5^^,^^43^^,^^44^^,^^45^^,^^46^^,^^47^^,^^48^^,^^49^^,^^50^^,^^51^^,^^52^^,^^53^^,^^54^^,^^55^ These studies revealed that different agonists interact with similar HCA_2_ amino acid residues, suggesting that ligand-receptor interactions along the entry path and residues outside the orthosteric pocket modulate ligand specificity and signaling outcomes. However, these structural studies have focused almost exclusively on human receptors and therefore do not explain how naturally occurring sequence variation across mammals translates into functional diversification of ligand recognition.

Despite the wealth of available structural data for HCA_2_ and its role in regulating metabolic and immune cell processes, its role in mammalian evolution remains unclear, particularly in adapting to changing metabolic conditions and microbial exposure.

We hypothesize that HCA_2_ has evolved under distinct selective pressures across mammalian lineages, potentially driving adaptive changes in ligand recognition. Such pressures can generate amino acid variation within ligand-binding sites, altering receptor pharmacology while preserving the conserved G-protein-coupling architecture.

Comparative genomic analyses have revealed that mammalian GPCRs frequently evolve under selective pressures driven by diet, physiology, and symbiotic microbial communities.^4^ Importantly, phylogenetic relatedness does not necessarily predict shared metabolite exposure, as diet, habitat, and microbiome composition can diverge substantially among closely related species. However, how such molecular changes translate into species-specific receptor function and which ecological or metabolic factors drive them remain unresolved for most metabolite-sensing GPCRs, including HCA_2_. We therefore tested how evolutionary sequence variation in HCA_2_ relates to species-specific metabolite environments and receptor pharmacology across mammals. A comprehensive functional comparison of mammalian HCA_2_ orthologs, integrating biochemical signaling, evolutionary conservation, and metabolomic context, has so far been lacking.

In this study, we combine pharmacological profiling, analysis of known 3D structural data, and evolutionary sequence analyses to elucidate the molecular determinants of HCA_2_ and HCA_3_ signaling diversity and investigate how cell culture conditions influence HCA_2_ signaling. To explore the evolutionary diversification of HCA_2_, we analyze over 200 mammalian orthologs and identify specific amino acid substitutions that correlate with altered ligand specificity. Structure-guided mutagenesis demonstrates that these substitutions causally reshape ligand selectivity. Functional assays across representative species reveal striking divergence, most notably in the African rhinoceros, where HCA_2_ has lost responsiveness to canonical HCA_2_ agonists but has gained sensitivity to HCA_3_-like ligands. Finally, using metabolomics of fecal extracts and site-directed mutagenesis, we show that HCA_2_ has undergone adaptive evolution, particularly at residues that shape the ligand-binding pocket, and suggest that these functional shifts are due to species-specific exposure to microbial metabolites. These findings define receptor-level evolutionary adaptation without invoking organismal fitness effects.

Together, our work provides the first phylogenetically broad, structure-guided functional analysis of mammalian HCA_2_, linking evolutionary sequence variation to ligand recognition shaped by microbiome-derived metabolites. We establish a comprehensive framework for understanding how HCA_2_ sequence variation and ecological factors influence receptor evolution, demonstrating lineage-specific functional diversification through adaptive modification of ligand-binding residues while preserving conserved signaling architecture. These findings illustrate how metabolite-sensing GPCRs can be evolutionarily tuned to species-specific metabolic environments. Importantly, this functional diversification is defined at the receptor level and does not imply organismal fitness effects or direct *in vivo* causality.

## Results

### Substantial differences in ligand potency, receptor selectivity, and signaling bias comparing human HCA_2_ and HCA_3_

**Figure 1:**
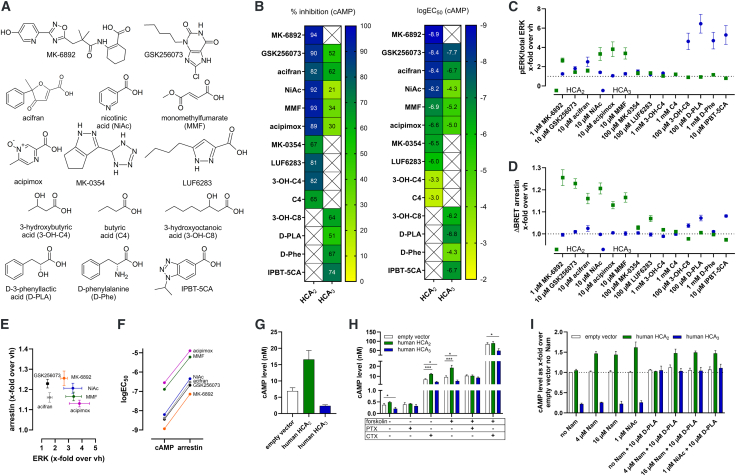
Variable signaling induced by agonists of human HCA_2_ and HCA_3_ (A) Structures and abbreviations of known HCA_2_ and HCA_3_ ligands. (B) Heatmaps showing percentage inhibition and logEC_50_ extrapolated from concentration-response curves in the presence of 2 μM forskolin measured in cAMP inhibition assays (mean, *n* = 4). (C) Phosphorylated and total ERK1/2 levels (*n* = 5) were determined. (D) For BRET analyses, cells were transfected with HCA_2_-mVenus and arrestin-3-Nluc (*n* = 6). BRET ratios were defined as acceptor emission/donor emission. (C and D) Data are shown as *x*-fold over vh (vehicle equals no agonist), mean ± SEM. (E) Data presented in (C and D) are plotted against each other to highlight the relationship of ERK signaling to arrestin-3 recruitment for the different ligands. (F) logEC_50_ values determined in cAMP inhibition assays (B) and BRET arrestin-3 recruitment assays (Table S1) are shown to visualize the shift in EC_50_ for all ligands for which HCA_2_-mediated arrestin-3 recruitment was determined. (G–I) Basal cAMP level determined for HCA_2_, HCA_3_, and empty vector-transfected CHO-K1 cells after 15 min incubation in HBSS with 2 μM forskolin (mean ± SEM, *n* = 3). (H) Cells were pre-incubated for 16 h with and without 100 ng/mL pertussis toxin (PTX) or cholera toxin (CTX). Statistical analyses were performed using unpaired two-tailed *t* tests. ∗*p* ≤ 0.05, ∗∗*p* ≤ 0.01, ∗∗∗*p* ≤ 0.001. (I) Cells were incubated overnight in nicotinamide-free custom-made medium without (no Nam) or with supplementation of Nam, NiAc, and/or D-PLA. Related to Table S1.

The closest paralog to human HCA_2_ is HCA_3_, both with known synthetic and natural ligands (Figure 1A). To compare their signaling profiles, we systematically examined the effects of various known ligands on cyclic adenosine monophosphate (cAMP) inhibition, extracellular signal-regulated kinases (ERKs) activation, and arrestin-3 recruitment (Figures 1B–1D).

MK-6892, so far the most potent and efficient HCA_2_-specific agonist, induced cAMP inhibition, activated ERK, and caused recruitment of arrestin-3 (Figures 1B–1D). NiAc, which was about 10,000-fold less potent at HCA_3_, showed HCA_2_-mediated activation of all three analyzed signaling components. GSK256073 activated HCA_3_ with 5-fold lower potency and half the efficacy compared to HCA_2,_ in contrast to a previously reported 100-fold lower potency.^37^ GSK256073 induced ERK activation via both HCA_2_ and HCA_3_ but arrestin-3 recruitment only via HCA_2_, a pattern also observed for acifran (Figures 1C and 1D). MMF and acipimox did not induce HCA_3_-dependent ERK signaling (Figures 1B–1D). MK-0354 and LUF6283 are both human HCA_2_-specific partial agonists; both recruited arrestin-3 (with a weaker effect for MK-0354) without ERK activation (Figures 1B–1D). In contrast to butyric acid (C4), the endogenous low-potent HCA_2_-specific ligand 3-OH-C4 slightly induced arrestin-3 recruitment (Figures 1B–1D). Human HCA_3_-specific ligands (3-hydroxyoctanoic acid (3-OH-C8), D-phenyllactic acid (D-PLA), D-phenylalanine (D-Phe), IPBT-5CA) showed no activity at human HCA_2_ (Figures 1A–1D). Both potent non-HCA_2_-specific ligands, GSK256073 and acifran, were inefficient in human HCA_2_-dependent ERK activation (Figures 1C and 1E). Concentration-response curves revealed that arrestin-3 recruitment potencies at HCA_2_ were about 50- to 70-fold lower than for cAMP inhibition (Figure 1F; Table S1). In summary, we comprehensively and functionally characterized the ligands that activate human HCA_2_ and HCA_3_.

### Standard cell culture medium modulates basal HCA_2_ signaling via G_i_-dependent sensitization

In forskolin-treated cells, HCA_2_-transfected cells showed increased basal cAMP levels compared to the control, while human HCA_3_ exhibited classical G_i_ protein-mediated reduction in cAMP levels (Figure 1G). Both effects were sensitive to the G_i_ protein inhibitor pertussis toxin (PTX), and not the G_s_ protein inhibitor cholera toxin (CTX) (Figure 1H). Dulbecco’s modified Eagle medium: nutrient mixture F12 (DMEM/F-12) medium contains 17 μM Nam, which itself has been reported not to bind to HCA_2_ or to act only as a very weak HCA_2_ agonist.^21^^,^^22^^,^^23^ Our data indicate that Nam, or trace NiAc present in the medium, accounts for the increased basal cAMP observed in HCA_2_-expressing cells via Gα_i/o_-coupled receptor-mediated heterologous sensitization of adenylyl cyclase.^56^ Using Nam-free medium abolished this effect, whereas adding 4 μM Nam restored it in HCA_2_ but not HCA_3_-transfected cells. Conversely, the addition of D-PLA selectively increased basal cAMP in HCA_3_-transfected cells (Figure 1I).

These findings confirm that standard culture media can contain low levels of HCA_2_ ligands that can modulate basal signaling. This is particularly relevant for comparative pharmacology, as baseline receptor activation by media components may differ across orthologs and influence apparent species differences in ligand potency. Because similar media are commonly used for receptor expression in structural studies, residual ligand occupancy should also be considered when interpreting HCA_2_ structures.

### Positional conservation analyses uncover ligand-binding adaptations in mammalian HCA_2_ orthologs with distinct HCA_3_-like substitutions in African rhinoceros

**Figure 2:**
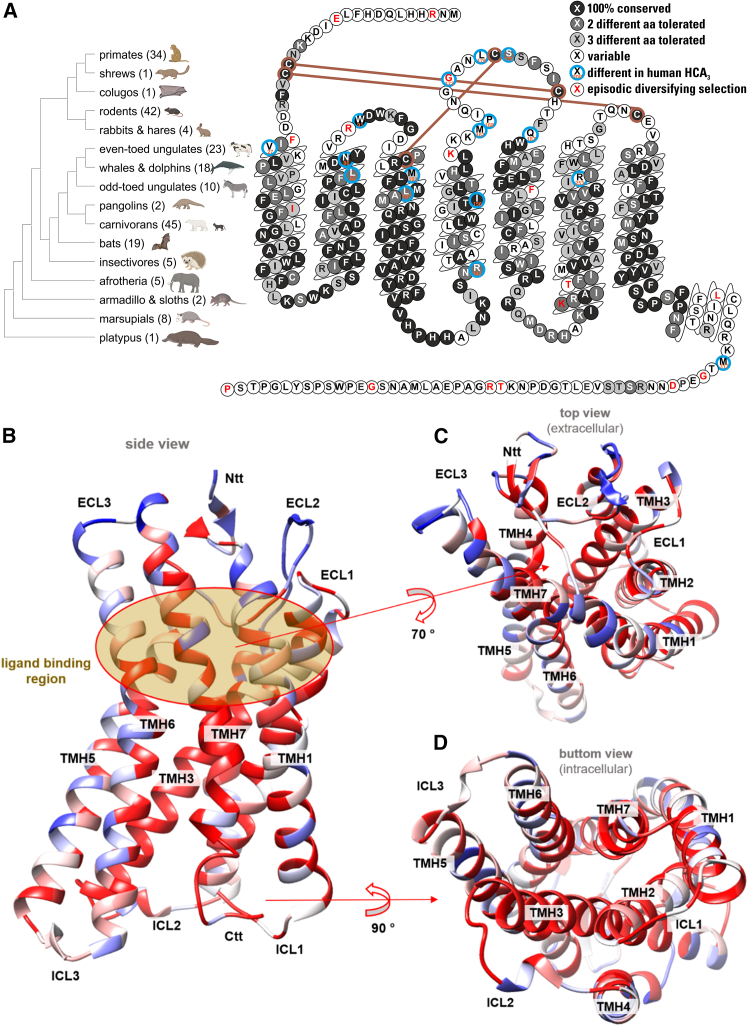
Conservation of mammalian HCA_2_ orthologs and comparison to human HCA_3_ (A) Phylogenetic tree with mammalian orders and the number of species (total, 220) included in conservation analyses visualized in the snake plot, and (B–D) mapped to a 3D structure using the software package Chimera.^57^ The color of the HCA_2_ backbone (PDB ID, 8j6q^44^) reflects the degree of conservation (red, highly conserved, blue, less/not conserved). (B) A brown circle indicates the principal ligand binding region, which shows high conservation (C), particularly for residues with side chains directed to the transmembrane domain core. Extracellular loop 3 (ECL3) and the N-terminal part of ECL2 are not conserved. (D) The G-protein interaction site (intracellular) is highly conserved, especially in intracellular loop 1 (ICL1) and ICL2. aa, amino acid; TMH, transmembrane helix; Ntt, N-terminal tail; Ctt, C-terminal tail. Related to Tables S2 and S3–S5.

Human HCA_2_ and HCA_3_ differ by only 16 amino acids and an extended C terminus, yet display distinct ligand signaling profiles, indicating that limited sequence variation can reshape ligand recognition.^16^ We therefore performed a positional conservation analysis across more than 200 mammalian HCA_2_ orthologs to identify evolutionary substitutions within the ligand-binding region (Figure 2A; Tables S2 and S3).

Notably, African rhinoceroses, white (*Ceratotherium simum*), and black rhinoceros (*Diceros bicornis*), exhibit substitutions at eight HCA_3_-specific sites, including key ligand-binding residues (Leu83^2.60^, Met103^3.28^, and Leu107^3.32^) and multiple positions in the extracellular loop 2 (ECL2; Met167, Pro168, Gly173, and Leu176) (Figure 2A; Table S3). Thus, HCA_2_ may have evolved under changing constraints to recognize a diverse range of ligands. Mixed effects model of evolution (MEME) analysis revealed several sites under episodic diversifying selection, including Gly173 (Figure 2A). Additionally, Phe25^1.30^, Arg90 (ECL1), and Lys164 (ECL2), located near divergent HCA_2_/HCA_3_ positions, also showed signatures of positive selection (Figure 2A). These patterns indicate lineage-specific diversification concentrated in ligand-binding regions.

To place these variations in structural context, residue conservation across mammalian orthologs was mapped onto available HCA_2_/HCA_3_ structures (Figures 2B–2D; Table S4).^5^^,^^43^^,^^44^^,^^45^^,^^46^^,^^47^^,^^48^^,^^49^^,^^50^^,^^51^^,^^52^^,^^53^^,^^54^^,^^55^

The principal orthosteric pocket is highly conserved, particularly residues projecting into the transmembrane core (Figure 2B). In contrast, ECL3 and the N-terminal portion of ECL2 show substantial variability, whereas residues implicated in G-protein interaction (ICL1 and ICL2) remain strongly conserved, indicating preservation of signaling architecture (Figures 2C and 2D; Table S5).

Based on this phylogenetic and structural analysis, we selected representative HCA_2_ orthologs spanning major mammalian clades and key ligand-binding substitutions for functional characterization, including marsupials, afrotherians, rodents, carnivores, odd- and even-toed ungulates, cetaceans, and primates, as well as human HCA_2_ and HCA_3_ (Figure S1A). This panel captures major evolutionary transitions in HCA_2_ ligand-binding sequence variation while maintaining conserved signaling interfaces.

### 3-OH-C4 and C4 more potently activate minke whale and opossum HCA_2_, while rhinoceros HCA_2_ is unresponsive to HCA_2_ agonists but activated by HCA_3_ ligands

**Figure 3:**
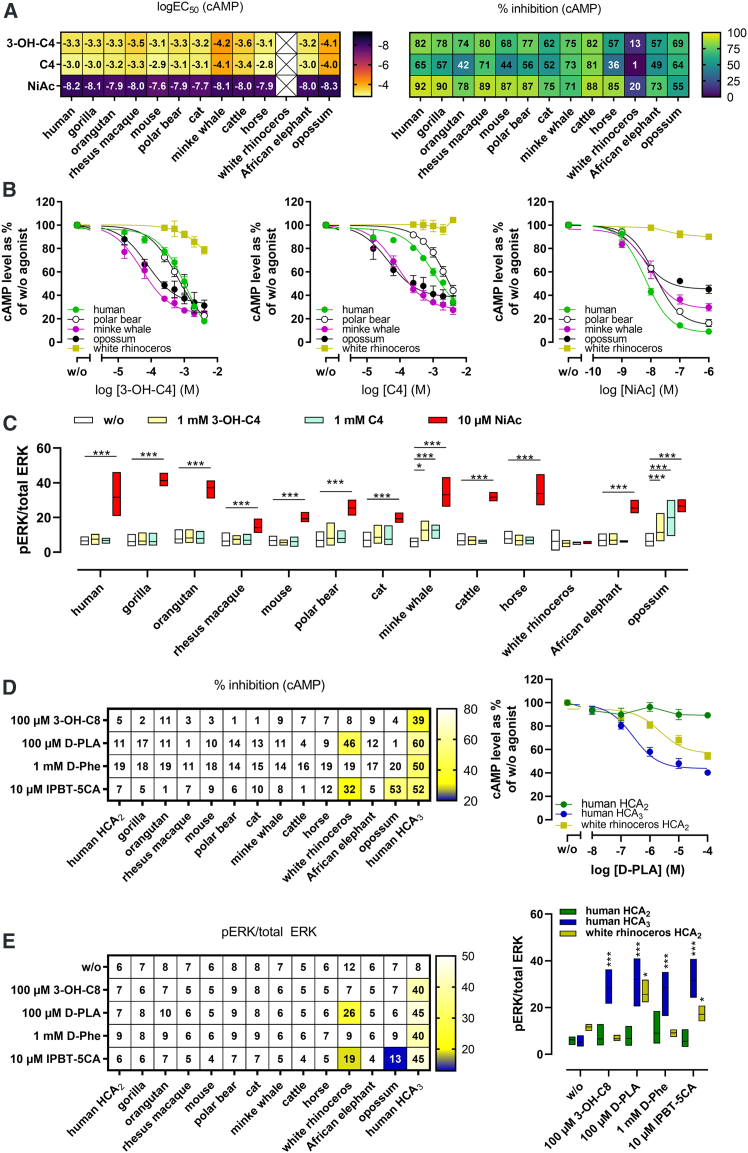
3-OH-C4 and C4 more potently activate minke whale and opossum HCA_2_, while rhinoceros HCA_2_ is unresponsive to HCA_2_ agonists but activated by HCA_3_ ligands Cells were transiently transfected with HCA_2_ orthologs or human HCA_3_. (A) Heatmaps showing logEC_50_ and percentage inhibition were extrapolated from concentration-response curves of 3-hydroxybutyric acid (3-OH-C4), butyric acid (C4), and nicotinic acid (NiAc) in cAMP inhibition assays performed in the presence of 2 μM forskolin (mean, *n* = 4). (B) Exemplary concentration-response curves. Data are shown as percentage of cAMP in the absence of agonist for each ortholog (w/o) as mean ± SEM. (C) Phosphorylated and total ERK1/2 levels (data shown as min to max, line at mean, *n* = 4). (D) Heatmap showing percentage inhibition determined in cAMP inhibition assays in the presence of 2 μM forskolin upon stimulation with indicated concentrations of 3-hydroxyoctanoic acid (3-OH-C8), D-phenyllactic acid (D-PLA), D-phenylalanine (D-Phe), or IPBT-5CA. Concentration-response curves upon stimulation with D-PLA. Data are shown as percentage of cAMP in the absence of agonist (w/o) for each ortholog as mean ± SEM, *n* = 4. (E) Heatmap showing phosphorylated over total ERK1/2 levels upon stimulation with indicated concentrations of 3-OH-C8, D-PLA, D-Phe, or IPBT-5CA. Comparison of pERK/total ERK for human HCA_3_ and rhinoceros HCA_2_ (data are shown as min to max, line at mean, *n* = 4). (C and E) Statistical analyses were performed using paired two-tailed *t* tests against w/o. ∗*p* ≤ 0.05, ∗∗*p* ≤ 0.01, ∗∗∗*p* ≤ 0.001. Related to Figure S1, Table S6.

Basal cAMP inhibition largely reflected HCA_2_ cell surface expression (Figure S1B). All orthologs were analyzed in the same cellular background, providing a common endogenous G-protein complement. Relative plasma membrane expression was quantified by cell-surface enzyme-linked immunosorbent assay (ELISA) under identical conditions for all orthologs analyzed, ensuring that pharmacological differences reflect receptor-intrinsic properties rather than variability in signaling context or expression levels. HCA_2_ from white rhinoceros, opossum, and cat showed lower surface expression and reduced basal cAMP levels, indicating higher basal G_i_ activation in these orthologs (Figure S1B). All HCA_2_ orthologs, except rhinoceros HCA_2,_ responded to 3-OH-C4 and C4 with notable differences in potency and efficacy (Figures 3A and 3B; Table S6). Notably, 3-OH-C4 and C4 were ∼10-fold more potent at HCA_2_ of minke whale and opossum, and these were the only orthologs showing ERK activation at a concentration of 1 mM (Figures 3A–3C; Table S6). NiAc induced cAMP inhibition with similar potency and efficacy, as well as ERK activation in all HCA_2_ orthologs except rhinoceros HCA_2_ (Figures 3A–3C; Table S6). Testing HCA_3_ agonists revealed that rhinoceros HCA_2_ gained responsiveness to D-PLA and IPBT-5CA, while opossum HCA_2_ also responded to IPBT-5CA (Figures 3D and 3E; Table S6). These findings demonstrate species-specific ligand recognition, with rhinoceros HCA_2_ showing extreme functional divergence driven by positional variation (Figure S1A; Table S6).

### African rhinoceros HCA_2_ shows strong positive selection, potentially driven by microbiome-derived metabolites, as HCA agonists are present in fecal samples

**Figure 4:**
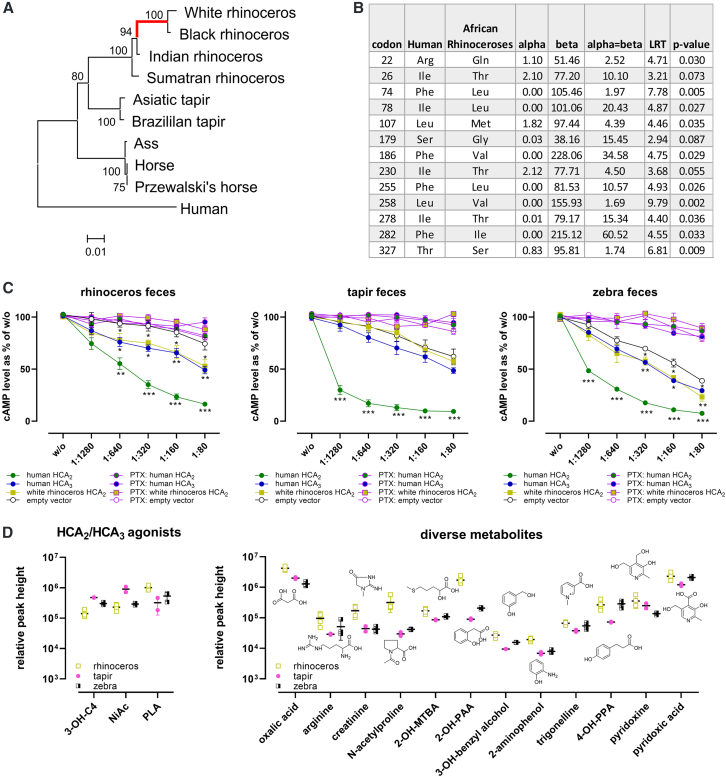
African rhinoceros HCA_2_ shows strong positive selection, potentially driven by microbiome-derived metabolites (A) Evolutionary history of odd-toed ungulate and human HCA_2_ was inferred using Maximum Likelihood (Kimura 2-parameter, MEGA 11). The tree (log likelihood = −2,483.78) shows branch lengths as substitutions per site, with bootstrap support indicated. RELAX revealed significant selection intensification in African rhinoceroses (K = 8.73, *p* ≤ 0.0001, LR = 14.90, highlighted in red). (B) Among the odd-toed ungulates HCA_2_ orthologs and human HCA_2_, shown in (A) FEL identified 13 sites (*p* ≤ 0.1) under diversifying positive selection in African rhinoceros HCA_2_ orthologs. Fecal samples from black rhinoceros (*n* = 4), Asiatic tapir (*n* = 2), and zebra (*n* = 3) were extracted, diluted, and (C) tested for cAMP inhibition in cells expressing human or rhinoceros HCA_2_, human HCA_3_, or empty vector, with or without pertussis toxin (100 ng/mL, 16 h). Data are shown as percentage of untreated controls (mean ± SEM, *n* ≥ 4). Statistical analyses were performed using unpaired two-tailed *t* tests, each against the respective empty vector control. ∗*p* ≤ 0.05, ∗∗*p* ≤ 0.01, ∗∗∗*p* ≤ 0.001. (D) HILIC-based metabolomics of all extracts identified relative levels of HCA_2_/HCA_3_ agonists and other metabolites; selected metabolites differing in concentrations are shown. 2-OH-MTBA, 2-hydroxy-4-(methylthio)butyrate; 2-OH-PAA, 2-hydroxy-phenylacetic acid; 4-OH-PPA, 4-hydroxy-phenylpropionic acid. Related to Figures S2 and S3.

Evolutionary analysis of odd-toed ungulate HCA_2_ orthologs revealed significant selection intensification in African rhinoceroses (K = 8.73, *p* ≤ 0.0001, LRT = 14.90), with 13 sites under positive selection (*p* ≤ 0.1), including Leu107^3.32^ (Figures 4A and 4B). We propose that microbiome-derived metabolites such as C4 and NiAc may be associated with these adaptations in rhinoceros HCA_2_. Therefore, fecal samples from rhinoceros, tapir, and zebra were collected, extracted with aqueous buffer to preferentially capture abundant soluble metabolites most likely to access luminal and systemic HCA_2_, and tested on human HCA_2_, HCA_3_, and rhinoceros HCA_2_ in cAMP assays (Figure 4C). PTX was used to determine the G_i_ protein dependence of the signal. Human HCA_2_ responded most weakly to rhinoceros extracts and most strongly to tapir extracts, consistent with the detected NiAc levels (Figures 4C and 4D). Only rhinoceros and zebra fecal extracts activated human HCA_3_ and rhinoceros HCA_2_, correlating with high levels of PLA and other metabolites (Figures 4C and 4D).

**Figure 5:**
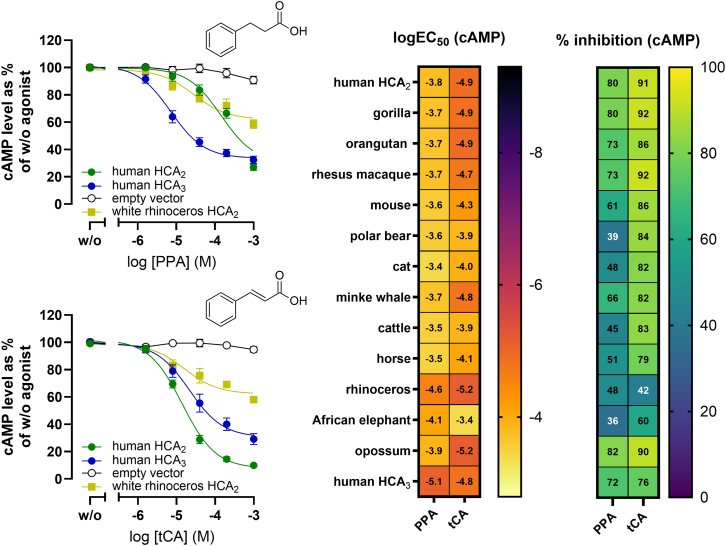
Phenylpropionic acid and *trans*-cinnamic acid are evolutionarily conserved HCA_2_ agonists Cells were transiently transfected with mammalian HCA_2_ orthologs, human HCA_3,_ or an empty vector control, and cAMP inhibition assays in the presence of 2 μM forskolin were performed upon stimulation with PPA and tCA. Data are shown as mean ± SEM (*n* = 3). Heatmaps are extrapolated from concentration-response curves and show percentage inhibition and logEC_50_ values. Related to Figures S3 and S4, Table S6.

Hydrophilic interaction liquid chromatography (HILIC)-based metabolomics analyses identified 83 metabolites, with rhinoceros samples showing low 3-OH-C4 levels but elevated concentrations of PLA and other metabolites that may contribute to the observed differences in activation patterns (Figures 4D and S2). Therefore, we tested all these compounds and other microbiota-derived metabolites for their agonistic activity at human HCA_2_ and HCA_3_, which reflect the ligand specificity differences seen in rhinoceros HCA_2_ (Figure S3). Most compounds were inactive but trigonelline selectively activated HCA_2_, while 2-hydroxy-4-(methylthio)butyrate (2-OH-MTBA), indolepropionic acid, and *trans*-indoleacrylic acid specifically activated HCA_3_ (Figure S3). Several aromatic acids activated both receptors, with phenylpropionic acid (PPA) and *trans*-cinnamic acid (tCA) inducing the highest reduction in cAMP (Figure S3). These data indicate that such aromatic acids can act as ligands across mammalian HCA_2_ orthologs, including rhinoceros HCA_2_. Indeed, PPA and tCA activated rhinoceros and all other mammalian HCA_2_ orthologs, with tCA generally being more potent (Figure 5; Table S6). However, the potency and efficacy of both ligands also varied across species (Figure 5; Table S6).

Together, these findings suggest that microbiome-derived metabolites, particularly aromatic acids such as PPA and tCA, may be linked to the evolutionary diversification of HCA_2_ in odd-toed ungulates, with rhinoceros HCA_2_ representing a striking example of adaptation to species-specific metabolic environments. To better understand the physiological roles of HCA_2_ across mammals, we next examined its tissue-specific RNA expression patterns, which are available and accessible for several species in the Bgee database, allowing for the comparison of mammalian transcriptomic data for some species (Figure S4).^58^^,^^59^ However, the white rhinoceros is a critically endangered species for which no RNA expression data are available.

### mRNA expression of HCA_2_ in different species

We investigated the distribution of HCA_2_ expression in immune, digestive, and other organ systems to assess conserved and species-specific functions.

HCA_2_ is expressed in immune-associated tissues, including lymph nodes, spleen, bone marrow, tonsils, and leukocytes (Figure S4). Furthermore, high expression is observed in the tongue of cattle, in the salivary glands of humans, sheep, and dogs, and in the esophagus of several species (Figure S4).

Notably, while HCA_2_ expression is low in the stomach, small, and large intestine of non-ruminants, it is elevated in the rumen of cattle, sheep, and goats, consistent with the role of microbial fermentation in plant digestion of ruminant animals (Figure S4).^60^ Other tissues for which relatively high HCA_2_ expression is found in several species of different orders include the olfactory bulb, the lung, skin, adipose tissue, and female reproductive tissues (Figure S4).

These expression patterns highlight a conserved role of HCA_2_ in immune-associated tissues across mammals. At the same time, the elevated levels in the rumen of ruminants point to a potential specialization in host-microbe interactions during plant digestion. Moreover, its presence in diverse epithelial and reproductive tissues suggests broader functions beyond immunity, possibly linking metabolic and barrier-related processes.

We further analyzed ligand preferences across mammalian HCA_2_ orthologs using available synthetic ligands.

### Divergent agonist responses of mammalian HCA_2_ orthologs reflect sequence variability in the ligand-binding pockets

**Figure 6:**
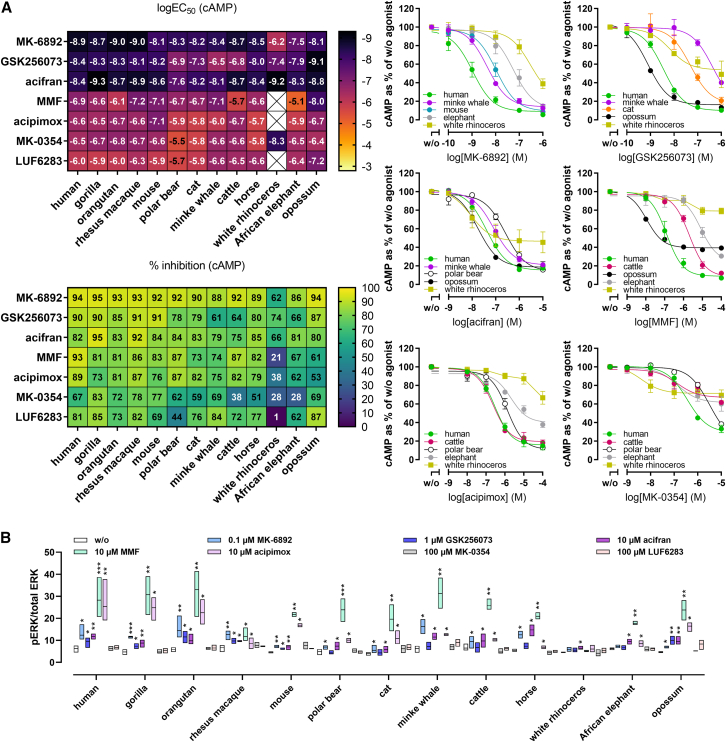
Synthetic HCA_2_ ligands exhibit variable ligand profiles at mammalian HCA_2_ orthologs Cells were transiently transfected with HCA_2_ orthologs or human HCA_3_. (A) Ligand-induced cAMP inhibition was determined in the presence of 2 μM forskolin. Exemplary concentration-response curves (data shown as percentage of cAMP in the absence of agonist for each ortholog (w/o) (mean ± SEM, *n* = 4), as well as heatmaps of logEC_50,_ and % inhibition extrapolated from these curves, are shown. (B) Phosphorylated and total ERK1/2 levels (data shown as min to max, line at mean, *n* = 4) upon stimulation with MK-6892, GSK256073, acifran, monomethylfumarate (MMF), acipimox, MK-0354, and LUF6283. Statistical analyses were performed using paired two-tailed *t* tests against w/o. ∗*p* ≤ 0.05, ∗∗*p* ≤ 0.01, ∗∗∗*p* ≤ 0.001. Related to Table S6.

Systematic profiling of mammalian HCA_2_ orthologs revealed striking species-specific differences in responses to synthetic agonists (Figure 6A; Table S6). MK-6892 activated rhinoceros HCA_2_ with ∼500-fold lower potency than human, and was also less potent at elephant HCA_2_, which is also reflected in a lack of MK-6892-induced ERK activation (Figure 6B). GSK256073 displayed higher potency at opossum HCA_2_ but was markedly weaker at polar bear, cat, minke whale, cattle, and rhinoceros HCA_2_ (Figure 6A; Table S6). Consequently, no GSK256073-mediated ERK activation was detected for these orthologs (Figure 6B). Acifran, although least potent at polar bear HCA_2_, strongly activated rhinoceros HCA_2_, while MMF and acipimox showed reduced potency at polar bear, cat, minke whale, horse and elephant orthologs, and did not activate rhinoceros HCA_2_ (Figure 6A; Table S6). Similarly, acifran stimulation resulted in ERK activation of all HCA_2_ orthologs, while MMF and acipimox failed to activate rhinoceros HCA_2_ (Figure 6B). MK-0354 activated rhinoceros HCA_2_ with high potency but low efficacy, whereas LUF6283 displayed high potency at opossum HCA_2_ but low potency at polar bear HCA_2_ (Figure 6A). MK-0354 and LUF6283 failed to induce ERK activation in any ortholog (Figure 6B).

**Figure 7:**
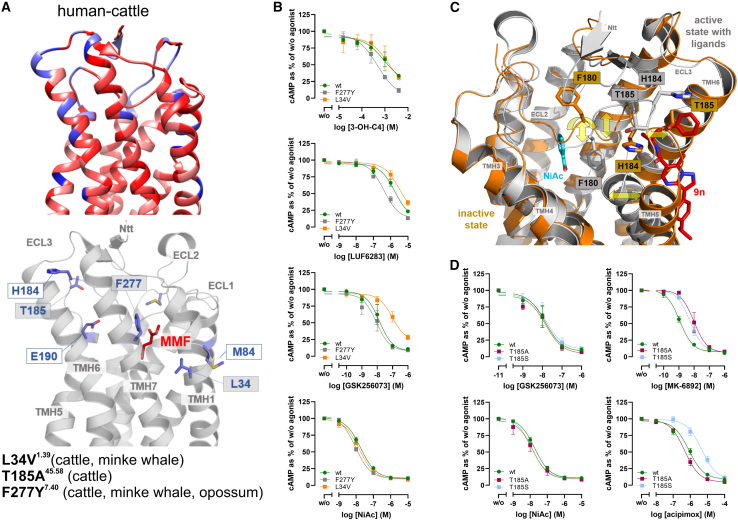
Conservation analyses at the active state 3D structure, combined with mutational analyses, identified key residues in the ligand-binding region that explain some of the species-specific functional differences (A) The human HCA_2_ sequence was compared with cattle HCA_2_, chosen due to its variation in responses to several ligands (Figure 6A). In addition, conservation was mapped onto the active-state structure (PDB-ID 8j6q), with red indicating high and blue low conservation (top). For analyses and representation of conservation degree, the software package *Chimera*^57^ was used. Variants chosen for experimental substitution and functional tests are highlighted with gray labels. Non-conserved residues between human and cattle HCA_2_ were visualized with exemplarily bound MMF from superimposed complexes. While many substitutions (e.g., M84, H184, and T185) are distant from the ligand, others, such as F277, are in direct proximity. (B and D) Cells were transiently transfected with human HCA_2_ wild-type (wt) or mutants, and ligand-induced cAMP inhibition was determined in the presence of 2 μM forskolin as indicated. Data are shown as percentage of cAMP in the absence of agonist for HCA_2_ mutant (w/o) as mean ± SEM (*n* = 3). (C) Active (PDB: 8j6q) versus inactive (PDB: 7zL9) HCA_2_ structures show marked differences in the C-terminal ECL2 and the adjoining TMH5 (helix-kink at P200^5^·^50^), while most of the extracellular parts overlap. Side-chain orientations differ, highlighting how substitutions or ligand binding can reshape this flexible region and impact orthosteric, allosteric, or bitopic ligand interactions. (A and C) Structural representations were generated using PyMol (Molecular Graphics System v.2.5.5, Schrödinger, LLC, New York, NY). ECL, extracellular loop; ICL, intracellular loop; TMH, transmembrane helix; Ntt, N-terminal tail; 3-OH-C4, 3-hydroxybutyric acid, NiAc, nicotinic acid. Related to Figure S5, Table S7.

Sequence conservation analysis across 220 mammalian orthologs identified hot spots in the ligand-binding region that may contribute to the observed differences in pharmacological properties (Figure S5; Table S7). Specifically, a comparison of cattle with human HCA_2_ revealed several differing positions located in the ligand-binding region (Figure 7A; Table S7). Thus, we generated the exemplary human HCA_2_ mutants F277Y^7.40^ and L34V^1.39^, as these substitutions occur only in minke whale, cattle, and opossum HCA_2_, of all orthologs functionally tested (Figure S1A). As for minke whale, cattle, and opossum HCA_2_, 3-OH-C4, and LUF6283 activated the mutant F277Y^7.40^ more potently (Figures 3A, 6A, and 7B). Similarly, GSK256073 less potently activated L34V^1.39^, as observed for minke whale and cattle HCA_2_, both of which have this amino acid exchange (Figures 6A and 7B; Figure S1A). However, neither mutant differed in potency for NiAc (Figure 7B).

Further, position T185^45.48^ in human HCA_2_, critical for allosteric ligand binding in ECL2 (Figure 7C; Table S7), is substituted by alanine in cattle and serine in elephant HCA_2_ (Figure S1A). The corresponding mutants T185A^45.58^ and T185S^45.48^ showed reduced MK-6892 potency, while GSK256073 and NiAc remained unaffected (Figure 7D). Acipimox potency was reduced in elephant but not cattle HCA_2_, consistent with our findings for T185S^45.48^ but not T185A^45.48^, which shows that position 185 affects the orthosteric binding site by modulating ECL2 conformation and dynamics (Figures 6A and 7D).

In summary, mutagenesis confirmed that substitutions at several positions fine-tune ligand potency and efficacy. These findings highlight that subtle sequence variations in the ligand-binding pocket are responsible for the striking functional diversity of HCA_2_ across mammals, shaping species-specific drug responses and revealing evolutionary adaptation at the receptor-ligand interface. Thus, the context-dependent interplay between HCA_2_ sequence variation and ligand activity offers a structural basis for the divergent pharmacological profiles observed across species.

## Discussion

Our study provides a functional, pharmacological, and evolutionary characterization of HCA_2_ across mammals, revealing species-specific differences in ligand potency, selectivity, and signaling that arise from sequence variation within ligand-binding sites. Integration of phylogenetic breadth with structure-guided functional analysis demonstrates that evolutionary divergence directly reshapes ligand recognition at HCA_2_, establishing a mechanistic link between microbiome-derived metabolites and receptor pharmacology across mammals.

### Differences in HCA_2_ and HCA_3_ ligand potency and selectivity

Consistent with previous structural and pharmacological studies, human HCA_2_ and HCA_3_ differ markedly in ligand preferences and downstream signaling bias (e.g., G-protein versus arrestin-3 or ERK pathways). Arrestin-3 recruitment was therefore assessed only for the human HCA_2_ and HCA_3_ reference receptors to define ligand signaling bias. NiAc is endogenously present in the plasma of healthy individuals at concentrations of ∼80 nM, primarily derived from dietary sources or microbial metabolism.^61^^,^^62^^,^^63^^,^^64^^,^^65^^,^^66^ The anti-dyslipidemic actions of NiAc at pharmacological concentrations of up to 200 μM are HCA_2_-independent, whereas the clinically limiting flushing response is mediated by HCA_2_ activation in skin cells.^27^ Synthetic HCA_2_ agonists remain of therapeutic interest for treating inflammatory conditions, and our analysis refines their signaling profiles. As previously shown, MK-0354 and LUF6283 acted as HCA_2_-selective partial agonists, primarily activating G_i_ protein signaling.^67^ However, in contrast to previous reports, neither ligand induced detectable ERK activation in our assays.^36^^,^^67^ Notably, MK-0354 promoted arrestin-3 recruitment despite prior reports suggesting a lack of receptor internalization, indicating that HCA_2_ can engage arrestin-dependent signaling at the plasma membrane without obligatory internalization (Figure 1). MK-6892 and GSK256073 have been proposed to favor G_i_ over arrestin pathways^52^ but our data did not support pronounced bias. Instead, MK-6892 showed the highest potency at HCA_2_ in both the G_i_ protein and the arrestin pathway (Figure 1).^50^ GSK256073 displayed lower HCA_2_ selectivity than previously described and, together with acifran, produced robust arrestin recruitment but comparatively weak HCA_2_-dependent ERK activation (Figure 1). Overall, these results refine ligand-specific signaling assignments and align with structural determinants of HCA_2_/HCA_3_ ligand recognition.

### Impact of culture conditions on HCA_2_ signaling

Our findings show that standard insect or eukaryotic cell culture media containing Nam or NiAc induce HCA_2_-mediated and G_i_-dependent heterologous sensitization of adenylyl cyclase (Figure 1).^56^ This effect can alter estimates of basal receptor activity or ligand potency, indicating that media-derived ligands can influence basal signaling and apparent pharmacology, particularly in cross-species comparisons. Media-derived NiAc may also occupy the receptor during protein expression, potentially influencing ligand occupancy in structural studies. NiAc binds HCA_2_ with nM affinity, and structural differences have primarily been observed only for higher affinity ligands such as MK-6892 and GSK256073. These considerations underscore the importance of controlling culture conditions when comparing HCA_2_ pharmacology and structure–function relationships across species.

### Species variation, adaptation, and ligand specificity

Across mammals, ligand-binding residues varied substantially while G-protein-interacting residues remained conserved. Consistently, HCA_2_ orthologs from cattle, minke whale, and opossum displayed increased potency for the endogenous ketone body 3-OH-C4 and microbial C4, indicating physiological activation at lower ligand concentrations (Figure 3). These findings demonstrate species-specific differences in HCA_2_ physiology at the interface of host metabolism and immunity that arise from evolutionary diversification of ligand recognition rather than from alterations in downstream signaling architecture.

### Unique evolutionary adaptation of HCA_2_ in the white rhinoceros

Rhinoceros HCA_2_ is unresponsive to canonical HCA_2_ agonists and instead recognizes HCA_3_-like ligands, representing the most divergent ortholog identified (Figures 3, 4, and 6). Because HCA_3_ is restricted to great apes, the HCA_3_-like pharmacological features observed in rhinoceros HCA_2_ likely reflect convergent evolution toward a similar ligand-recognition profile rather than coordinated diversification between paralogs, highlighting independent evolutionary solutions to similar metabolite environments. This shift correlates with HCA_3_-like substitutions under positive selection and with microbiome-derived metabolites detected in rhinoceros fecal extracts, supporting receptor-level adaptation to species-specific metabolite environments without invoking organismal fitness effects.

Two evolutionary scenarios could explain this divergence. First, rhinoceros may no longer require HCA_2_ to sense fasting-associated metabolites such as 3-OH-C4 levels, potentially because this function has shifted to another GPCR, allowing HCA_2_ to specialize toward microbiome-derived ligands such as PLA. Alternatively, a conserved, yet unidentified endogenous ligand may activate HCA_2_ across mammals, while residues not required for its recognition diversified under species-specific dietary and microbial pressures. Consistent with this view, fecal metabolite profiles differ markedly among odd-toed ungulates (rhinoceros, tapir, and zebra) and correlate with distinct activation patterns of rhinoceros HCA_2_, human HCA_2_, and HCA_3_ (Figures 4, S2, and S3).

These features are consistent with the rhinoceros hindgut-fermenting herbivorous physiology, which shapes a distinct microbial metabolite environment rich in plant- and fermentation-derived aromatic compounds, which represent plausible selective drivers of the HCA_3_-like ligand recognition observed in rhinoceros HCA_2_.^68^^,^^69^^,^^70^ Similar diet- and microbiome-associated diversification of ligand-binding residues has been reported for other metabolite- and chemosensory-GPCR families, supporting evolutionary tuning of receptor specificity to ecological niche.^71^

### Microbial aromatic amino acid metabolites as conserved HCA_2_ agonists

Phenylpropanoid metabolites such as PPA and tCA activated all HCA_2_ orthologs, including rhinoceros HCA_2_ and human HCA_3_, indicating that aromatic microbial metabolites represent conserved ligands across mammalian HCA_2_ orthologs (Figure 5). Both ligands differed in potency and efficacy, with PPA generally less potent and efficacious than tCA across species.

PPA is not produced by mammalian metabolism but by Firmicutes and Bacteroidetes.^72^ In humans, circulating levels are in the micromolar range, and fecal concentration reaches ∼515 μM.^73^^,^^74^^,^^75^ Although previously reported as HCA_3_-selective, we show that PPA also activates HCA_2_ (EC_50_ ∼ 250 μM), albeit ∼25-fold less potently than at HCA_3_ (Figure 5), suggesting that the reported PPA-mediated protection against acetaminophen hepatotoxicity may involve both receptors.^6^^,^^72^

Furthermore, tCA, a plant- and microbiome-derived compound with documented antioxidant and anti-inflammatory properties, is likewise abundant in intestinal environments and activated both HCA_2_ and HCA_3_ across species (Figure 5).^74^^,^^76^^,^^77^^,^^78^^,^^79^^,^^80^^,^^81^^,^^82^^,^^83^^,^^84^ Together, these data identify phenylpropanoid microbial metabolites as evolutionarily conserved HCA_2_ ligands linking microbiome metabolism to receptor diversification.

### Molecular basis of functional divergence of mammalian HCA_2_ orthologs

To link the observed species-specific differences in ligand responses to underlying sequence variation, we next examined substitutions within the ligand-binding pocket and tested their functional impact.

Using structure-guided mutagenesis, we identified ligand-binding pocket substitutions that substantially alter ligand potency and efficacy. These residues map to regions previously implicated in HCA ligand recognition and align with structural studies distinguishing HCA_2_ and HCA_3_ ligand selectivity. Because these substitutions were experimentally tested in a common receptor background, the observed potency shifts establish direct causal relationships between sequence variation and pharmacological divergence. While these data establish causal effects on ligand potency, the precise structural mechanisms by which these substitutions reshape the binding pocket remain to be resolved and will require dedicated structural or computational analyses. Although available structures focus largely on human receptors, our analysis shows that naturally occurring mammalian HCA_2_ orthologs harbor substitutions at the same or analogous positions that correlate with altered pharmacology.

Notably, the F277Y^7.40^ substitution in human HCA_2_ shifted ligand preferences toward those of cattle, minke whale, and opossum (Figures 6 and 7). Its fixation in even-toed ungulates and whales, together with independent occurrence in marsupials, indicates recurrent selection in distant mammalian clades, consistent with convergent pressures on HCA_2_ function imposed by diet, metabolism, or microbiome environments. Concordance between phylogenetic distribution of substitutions and functional effects across representative orthologs further indicates that the sampled panel captures key evolutionary transitions in mammalian HCA_2_ diversification. Similarly, the T185S^45.48^ (ECL2) substitution, also present in African elephant HCA_2,_ reduced the potencies of MK-6892 and acipimox in the human receptor background (Figures 6 and 7).

Together, these results identify positions within the orthosteric and extended binding pockets as recurrent “hot spots” for adaptive diversification, whereas residues that mediate G-protein coupling remain highly conserved across mammals. This pattern mirrors broader GPCR evolution, in which ligand-binding regions diversify under selective pressure while signaling architecture is preserved. Thus, species-dependent differences in surrogate ligand potency likely reflect evolutionary tuning of HCA_2_ toward distinct endogenous metabolite repertoires shaped by diet and microbiome context.

### Physiological and translational implications

Our findings indicate that species-specific HCA_2_ pharmacology must be considered in preclinical ligand evaluation. Compounds potent at human HCA_2_ can differ substantially in potency, efficacy, or selectivity across mammalian orthologs, creating risks for cross-species translation and interpretation of efficacy or safety. Similarly, structural or pharmacological studies assuming conserved ligand recognition across species may not reflect *in vivo* receptor function.

The data further support the concept that microbiome-derived metabolites shape GPCR ligand specificity. While lactic acid bacteria-derived ligands regulate HCA_3_ signaling in humans, our results extend this principle to mammalian HCA_2_, identifying aromatic microbial metabolites as plausible selective pressures underlying rhinoceros receptor divergence. These observations are consistent with co-adaptation of diet, microbiome composition, and metabolite-sensing receptor specificity in host signaling networks.

Finally, we show that common culture media components can act as low-level HCA_2_ agonists, potentially introducing baseline receptor activation in functional or structural assays. This is particularly relevant for metabolite-sensing GPCRs whose endogenous ligands are present in standard media formulations. Together, these findings emphasize that species-specific GPCR pharmacology arises from evolutionary tuning of ligand recognition and should be considered in animal models and cross-species translation of receptor-targeted therapeutics.

In conclusion, our data demonstrate that HCA_2_ exhibits greater evolutionary functional diversification in receptor-level ligand recognition than previously appreciated. Sequence variation concentrated in the ligand-binding pocket, likely shaped by evolutionary pressures such as exposure to microbiome-derived metabolites, drives remarkable divergence in ligand potency and selectivity across mammals while preserving conserved G-protein coupling interfaces. These findings demonstrate that metabolite-sensing GPCRs can undergo lineage-specific functional tuning through adaptive modification of ligand recognition without altering core signaling architecture. This evolutionary plasticity has important implications for receptor biology and cross-species pharmacology and highlights how host-microbiome metabolic interactions can shape GPCR function. Importantly, these findings define receptor-level functional diversification without implying direct effects on organismal fitness or direct *in vivo* causality.

### Limitations of the study

While our ortholog panel spans major mammalian orders, additional species occupying specialized metabolic niches, such as highly specialized herbivores or animals adapted to extreme environments, may reveal further HCA_2_ divergence.

Although we observe correlations between microbiome metabolite profiles and receptor properties, direct *in vivo* evidence linking ligand exposure to receptor function in species such as rhinoceros remains to be established. Fecal metabolite profiling was based on aqueous extraction, which enriches soluble metabolites most relevant to intestinal and systemic HCA_2_ exposure but may underrepresent less soluble microbiome products. The present study, therefore, focuses on receptor-level molecular mechanisms, for which comparative pharmacology and structure-guided mutagenesis provide sufficient resolution to infer functional diversification. Structural data for non-human HCA_2_ orthologs, along with a broader analysis of downstream signaling bias, would further refine the understanding of ligand-receptor co-adaptation across mammals.

## Resource availability

### Lead contact

Requests for further information and resources should be directed to and will be fulfilled by the lead contact, Claudia Stäubert (claudia.staeubert@medizin.uni-leipzig.de).

### Materials availability

All unique plasmids generated in this study are available from the lead contact.

### Data and code availability

This article analyzes existing, publicly available data. Therefore, for all sequences of mammalian HCA_2_ orthologs, NCBI GenBank database accession numbers are listed in Table S2. This article does not report original code. Any additional information required to reanalyze the data reported in this article is available from the lead contact on request.

## Acknowledgments

This work was supported by 10.13039/501100001659Deutsche Forschungsgemeinschaft through CRC1423, project number 421152132, subproject C06 (to C.S.) and subprojects A01/Z03 (to P.S.) and through Germany’s Excellence Strategy – EXC 2008/1 (UniSysCat) – 390540038 (to G.K. and P.S.). C.S. was supported by the 10.13039/501100001659Deutsche Forschungsgemeinschaft – project number 407707190. The funders had no role in study design, data collection and analysis, decision to publish, or preparation of the manuscript. We want to thank the contributors of species samples (Table S9) and Frank Meyer from Zoo Leipzig for the valuable information on the lifestyle and specialties of rhinoceroses. The Swedish Metabolomics Center, Umeå, Sweden (www.swedishmetabolomicscentre.se), Anders Nordström, Annika Johansson, Elin Näsström, and Hans Stenlund are acknowledged for metabolic profiling by HILIC-LC-MS. We acknowledge support from Leipzig University for open access publishing.

## Author contributions

Conceptualization, formal analysis, data curation, and writing – original draft, F.B. and C.S.; methodology, F.B., P.K., G.K., P.S., and C.S.; validation, P.K. and C.S.; investigation, F.B., P.K., and C.S.; resources, C.S.; writing – review and editing and visualization, F.B., G.K., P.S., and C.S.; structural investigations, validation, conclusions, and visualizations, G.K. and P.S.; supervision and project administration, C.S.; funding acquisition, P.S. and C.S. All authors discussed the results and implications and commented on the manuscript at all stages. All authors read and approved the final manuscript.

## Declaration of interests

The authors declare no competing interests.

## STAR★Methods

### Key resources table

**Table:** 

REAGENT or RESOURCE	SOURCE	IDENTIFIER
**Antibodies**		

Anti-HA-Peroxidase, High Affinity from rat IgG1 monoclonal	Sigma-Aldrich	12013819001; RRID: AB_390917

**Biological samples**		

Fecal samples Rhinoceros	Zoo Leipzig	N/A
Fecal samples Tapir	Zoo Leipzig	N/A
Fecal samples Zebra	Zoo Leipzig	N/A
Genomic DNA see Table S9 for species	see Table S9 for sources	N/A

**Bacterial and virus strains**		

NEB 5-alpha competent *E.coli*	NEB	C2987

**Chemicals, peptides, and recombinant proteins**		

10x HBSS	Thermo Fisher Scientific	14065056
1x D-PBS	Thermo Fisher Scientific	14190144
2-aminophenol	Sigma-Aldrich	a71301 (CAS: 95-55-6)
2-hydroxy-4-(methylthio)butyric acid	MedChemExpress	HY-116688 (CAS: 583-91-5)
2-hydroxy-phenylacetic acid	Sigma-Aldrich	H49804 (CAS: 614-75-5)
3-hydroxybenzoic acid	Sigma-Aldrich	H20008 (CAS: 99-06-9)
3-hydroxybenzyl alcohol	Sigma-Aldrich	H20601(CAS: 620-24-6)
3-hydroxybutyrate	Sigma-Aldrich	298360 (CAS: 13613-65-5)
3-hydroxyoctanoate	Santa Cruz	sc-214136 (CAS: 88930-08-9)
3-hydroxy-phenylacetic acid	Sigma-Aldrich	H49901 (CAS: 621-37-4)
3-isobutyl-1-methylxanthine (IBMX)	Sigma-Aldrich	I5879 (CAS: 28822-58-4)
3-phenylpropionic acid	Sigma-Aldrich	W288918 (CAS: 501-52-)
4-hydroxybenzoic acid	Sigma-Aldrich	H20059 (CAS: 99-96-7)
4-hydroxy-phenylacetic acid	Sigma-Aldrich	H50004 (CAS: 156-38-7)
4-hydroxy-phenylpropionic acid	Sigma-Aldrich	H52406 (CAS: 501-97-3)
Acifran	Hölzel Diagnostika Handels GmbH	HY-107579 (CAS: 72420-38-3)
Acipimox	Sigma-Aldrich	A7856 (CAS: 51037-30-0)
Bovine serum albumin (BSA)	Sigma-Aldrich	A3294 (CAS: 9048-46-8)
Butyrate	Sigma-Aldrich	B5887 (CAS: 156-54-7)
Cholera toxin	Sigma-Aldrich	C8052 (CAS: 9012-63-9)
Citric acid	Sigma-Aldrich	251275 (CAS: 77-92-9)
Creatinine	Sigma-Aldrich	C4255 (CAS: 60-27-5)
Deoxynucleotide (dNTP) Solution Set	NEB	N0446
D-phenylalanine	Carl Roth	7886.1 (CAS: 673-06-3)
D-phenyllactic acid	Sigma-Aldrich	376906 (CAS: 7326-19-4)
Dulbecco’s Modified Eagle Medium (DMEM)	Thermo Fisher Scientific	41966029
Dulbecco’s Modified Eagle Medium (DMEM)/ F-12	Thermo Fisher Scientific	21041025
Fetal bovine serum (FBS)	Thermo Fisher Scientific	10270106
Formaldehyde	Sigma-Aldrich	F8775 (CAS: 50-00-0)
Forskolin	Sigma-Aldrich	F3917 (CAS: 66575-29-9)
GSK256073	Hölzel Diagnostika Handels GmbH	HY-119222 (CAS: 862892-90-8)
H_2_O_2_	Sigma-Aldrich	1.08600 (CAS: 7722-84-1)
HCl	Sigma-Aldrich	258148 (CAS: 7647-01-0)
HEPES (1M)	Thermo Fisher Scientific	15630056
Imidazole propionic acid	Sigma-Aldrich	77951 (CAS: 1074-59-5)
Indolepropionic acid	Molport	Molport-000-139-896 (CAS: 830-96-6)
IPBT-5CA	Biomol GmbH	Cay15223 (CAS: 306935-41-1)
L-arginine	Carl Roth	1655.2 (CAS: 74-79-3)
Lipofectamine 2000	Thermo Fisher Scientific	11668019
LUF6283	Hölzel Diagnostika Handels GmbH	HY-100976 (CAS: 92933-48-7)
MK-0354	Hölzel Diagnostika Handels GmbH	HY-13008 (CAS: 851776-28-8)
MK-6892	Hölzel Diagnostika Handels GmbH	HY-10680 (CAS: 917910-45-3)
Monomethylfumarate	Sigma-Aldrich	651419 (CAS: 2756-87-8)
N-Acetyl-L-proline	Sigma-Aldrich	A0783 (CAS: 68-95-1)
Na_2_HPO_4_ · 2 H_2_O	Sigma-Aldrich	S9763 (CAS: 7558-79-4)
Na_2_SO_3_	Sigma-Aldrich	S0505 (CAS: 7757-83-7)
Nicotinamide	Sigma-Aldrich	47865-U (CAS: 98-92-0)
Nicotinic acid	Sigma-Aldrich	N0761 (CAS: 59-67-6)
o-phenylenediamine	Sigma-Aldrich	P23938 (CAS: 95-54-5)
Opti-MEM	Thermo Fisher Scientific	31985070
Oxalic acid	Sigma-Aldrich	194131 (CAS: 144-62-7)
Penicillin-Streptomycin (5.000 U/ml)	Thermo Fisher Scientific	15140122
Pertussis toxin	Sigma-Aldrich	516560 (CAS: 70323-44-3)
Phenylacetic acid	Sigma-Aldrich	P16621 (CAS: 103-82-2)
Poly-L-lysine solution (0.1% (w/v) in H_2_O)	Sigma-Aldrich	P8920 (CAS: 25988-63-0)
pyridoxine	Sigma-Aldrich	P5669 (CAS: 65-23-6)
pyridoxic acid	Sigma-Aldrich	P9630 (CAS: 82-82-6)
Q5 High-Fidelity DNA Polymerase	NEB	M0491
Roswell Park Memorial Institute (RPMI) custom-made niacin-, glutamin-free medium	Cell Culture technologies	N/A
Trans-cinnamic acid	Sigma-Aldrich	C80857 (CAS: 140-10-3)
Trans-indoleacrylic acid	Sigma-Aldrich	I3807 (CAS: 29953-71-7)
Trans-urocanic acid	Molport	Molport-006-833-341 (CAS: 3465-72-3)
Trigonelline hydrochloride	Sigma-Aldrich	t5509 (CAS: 6138-41-6)
Tris/HCl	Sigma-Aldrich	10812846001 (CAS: 1185-53-1)
Triton X-100	Sigma-Aldrich	T8787 (CAS: 9036-19-5)
Trypsin-EDTA (0.25%), phenol red	Thermo Fisher Scientific	25200056
Tween 20	Sigma-Aldrich	P1379 (CAS: 9005-64-5)
UltraPure 0.5 M EDTA, pH 8	Thermo Fisher Scientific	15575020
Versene	Thermo Fisher Scientific	15040033

**Critical commercial assays**		

AlphaScreen cAMP assay kit	PerkinElmer Life Sciences	6760635M
Alpha SureFire Ultra Multiplex Phospho/Total ERK1/2 Assay Kit	PerkinElmer Life Sciences	MPSU-PTERK-M10K
NanoBRET NanoGlo substrate	Promega	N1572

**Deposited data**		

NCBI database accession numbers of mammalian *HCAR2* orthologs	see Table S2	NCBI Nucleotide, RRID: SCR_004860

**Experimental models: Cell lines**		

CHO-K1 (passage #3 to max. #40)	ATCC	CCL-61; RRID: CVCL_0214
HEK293-T (passage #3 to max #40)	ATCC	CRL-3216; RRID: CVCL_0063

**Oligonucleotides**		

see Table S8 for sequences	SeqLab	N/A

**Recombinant DNA**		

HA-/FLAG-tagged human-HCAR2 in pcDps	Peters et al.^16^	N/A
HA-/FLAG-tagged human-HCAR2 L34V in pcDps	This paper	N/A
HA-/FLAG-tagged human-HCAR2 T185A in pcDps	This paper	N/A
HA-/FLAG-tagged human-HCAR2 T185S in pcDps	This paper	N/A
HA-/FLAG-tagged human-HCAR2 F277Y in pcDps	This paper	N/A
HA-/FLAG-tagged human-HCAR3 in pcDps	Peters et al.^16^	N/A
HA-/FLAG-tagged gorilla-HCAR2 in pcDps	Peters et al.^16^	N/A
HA-/FLAG-tagged orangutan-HCAR2 in pcDps	Peters et al.^16^	N/A
HA-/FLAG-tagged rhesus macaque-HCAR2 in pcDps	This paper	N/A
HA-/FLAG-tagged mouse-HCAR2 in pcDps	Peters et al.^16^	N/A
HA-/FLAG-tagged polar bear-HCAR2 in pcDps	This paper	N/A
HA-/FLAG-tagged cat-HCAR2 in pcDps	This paper	N/A
HA-/FLAG-tagged minke whale-HCAR2 in pcDps	This paper	N/A
HA-/FLAG-tagged cattle-HCAR2 in pcDps	This paper	N/A
HA-/FLAG-tagged horse-HCAR2 in pcDps	This paper	N/A
HA-/FLAG-tagged white rhinoceros-HCAR2 in pcDps	This paper	N/A
HA-/FLAG-tagged African elephant-HCAR2 in pcDps	This paper	N/A
HA-/FLAG-tagged opossum-HCAR2 in pcDps	This paper	N/A
pcDps	Okayama et al.^85^	N/A
human-HCAR2-mVenus in EGFP-N1	This paper	N/A
human-HCAR3-mVenus in EGFP-N1	This paper	N/A
human arrestin-3-nanoluciferase in EGFP-N1	Liebing et al.^86^	N/A

**Software and algorithms**		

aBSREL (adaptive Branch-Site Random Effects Likelihood)	Smith et al.^87^	https://www.datamonkey.org/absrel
Bioedit Sequence Alignment Editor 7.0.9	Hall et al.^88^	
Biorender	Biorender	https://biorender.com/
Datamonkey	Weaver et al.^89^	https://www.datamonkey.org
DNASTAR Lasergene	DNASTAR Lasergene	https://www.dnastar.com/software/lasergene/
FEL	Kosakovsky et al.^90^	https://www.datamonkey.org/fel
GraphPad Prism Version 10	Graphpad Software	https://www.graphpad.com/scientific-software/prism/
MEGA11: Molecular Evolutionary Genetics Analysis Version 11	Tamura et al.^91^	https://megasoftware.net/home
MEME (Mixed Effects Model of Evolution)	Murrell et al.^92^	https://www.datamonkey.org/meme
PyMol Molecular Graphics System Version 2.5.5	Schrödinger, LLC, New York, NY	https://www.pymol.org
RELAX	Wertheim et al.^93^	https://www.datamonkey.org/relax
SnapGene Viewer	SnapGene	https://www.snapgene.com/snapgene-viewer

**Other**		

Maxi-Sorp flat bottom 96-well plates	Thermo Fisher Scientific	44-2404-21
OptiPlate-384, White Opaque 384-well Microplate	PerkinElmer Life Sciences	6007290
Multimode multilabel plate reader able to detect Perkin Elmer Alpha Technology	PerkinElmer Life Sciences	EnVision 2104 multilabel plate reader with barcode #444 mirror (cat#2101-4010), barcode #244 AlphaScreen 570/100 filter (cat #2100-5710) equipped with laser (excitation: 680 nm) by default)
Plate Reader to measure absorbance at 492 nm and 620 nm	Tecan	Tecan-Reader Infinite M Nano
Orbital shaker	Heidolph	Heidolph Titramax 1000
Shaker with 37 °C incubator hood	Edmund Bühler	Edmund Bühler shaker SM30 with incubator hood TH30
Overhead shaker	Heidolph	Heidolph Reax 2
Multi-dispensing multi-channel pipettes	Gilson /Brandt / Thermo	Pipetman G Multichannel P12x200G (12 channel) Tranferpette S-12, (0.5 – 10 μl) Finnpipette Novus (16 channel)
Aspiration system	Biovac	Biovac 106 with 2 l glass bottle

### Experimental model and study participant details

#### Cell lines

The Chinese hamster ovary cell line CHO-K1 (ATCC CCL-6, female) and the human embryonic kidney cell line HEK293-T (ATCC CRL-3216, female) were acquired from the American Type Culture Collection. CHO-K1 cells were grown in Dulbecco’s Modified Eagle Medium: Nutrient Mixture F-12 (DMEM/F12) and HEK293-T cells in Dulbecco’s Modified Eagle medium (DMEM), both supplemented with 10 % fetal bovine serum (FBS), 10,000 U/mL penicillin, and 10,000 μg/ml streptomycin. Cells were maintained at 37 °C in a humidified 5 % CO_2_ incubator. For transient transfection, Lipofectamine 2000 (Thermo Scientific) was used according to the manufacturer’s instructions.

#### Ethics statement

All fecal samples were collected noninvasively from the ground during routine animal husbandry procedures by the professional zookeepers at Zoo Leipzig. No animal activities or interventions were involved.

### Method details

#### *HCAR2* ortholog identification

*HCAR2* sequences of various mammalian species were obtained using NCBI Blast (accession numbers are listed in Table S2).

To generate expression plasmid encoding selected mammalian *HCAR2* full-length orthologs, primer pairs (Table S8) were used to amplify specific *HCAR2* sequences from genomic DNA samples (Table S9).^16^^,^^94^ Human, gorilla, orangutan, and mouse *HCAR2* have been previously described.^16^

PCR reactions were performed with Q5 polymerase under variable annealing and elongation conditions following the manufacturer’s protocol. The full-length *HCAR2* orthologs were inserted into the mammalian expression vector pcDps^85^ and epitope-tagged with an N-terminal hemagglutinin (HA) epitope and a C-terminal FLAG-tag by a PCR-based overlapping fragment approach. In addition, *HCAR2* was C-terminally tagged with mVenus. The identity of all constructs and the correctness of all PCR-derived sequences were confirmed by sequencing (Seqlab). All primers used for cloning are listed in Table S8.

#### Sequence alignments and datamonkey analyses

*HCAR2* nucleotide sequences of 220 mammalian species were aligned to infer a phylogenetic tree. All nucleotide alignments were generated with the Clustal W algorithm (Bioedit Sequence Alignment Editor 7.0.9),^88^^,^^95^ followed by manual trimming where gaps were deleted. The phylogenetic evolutionary history was inferred by the Maximum Likelihood method based on the General Time Reversible model using MEGA11.^91^ The resulting tree is shown in Figure S6. In addition, this nucleotide alignment was uploaded to the Datamonkey web server to be analyzed for evolutionary signatures.^89^ MEME (Mixed Effects Model of Evolution) was performed to detect episodic/positive selection at individual sites.^92^ aBSREL (adaptive Branch-Site Random Effects Likelihood) was applied to detect positive selection at individual branches.^87^ RELAX tests were used to analyze for selection relaxation or intensification along the specified set of branches^93^ and FEL (Fixed Effects Likelihood)^90^ were used to analyze whether individual sites were subject to pervasive (across the whole phylogeny) positive or purifying selection.

#### Transient transfection for functional assays

For transient transfection, Lipofectamine 2000 (Thermo Scientific) was used according to the manufacturer’s instructions. For cAMP assay and ELISA analyses, CHO-K1 cells were split into 25 cm^2^-cell culture flasks (0.9 x 10^6^ cells/flask) and transfected with a total amount of 3 μg of plasmid the following day. For arrestin-3 recruitment analyses, HEK293-T cells were seeded into 75 cm^2^-cell culture flasks (3.5 x 10^6^ cells/flask) and transfected the following day with 3.3 μg plasmid encoding C-terminally mVenus-tagged human *HCAR2* and 0.7 μg arrestin-3-Nanoluciferase (Nluc) construct.

#### ALPHAScreen cAMP assay and cell-surface ELISA

ALPHAScreen cAMP inhibition assays and ELISA-based analyses of cell surface expression of N-terminal HA-tagged receptor constructs were performed as exhaustively described in this protocol.^96^ Cyclic AMP content of cell extracts was determined using the ALPHAScreen cAMP Detection Kit (Revvity). CHO-K1 cells were incubated in serum-free media overnight. Cells were stimulated for 15 min at 37 °C in HBSS with 1 mM 3-isobutyl-1-methylxanthine and 2 μM forskolin containing different agonists in different concentrations. Reactions were stopped by aspiration of the stimulation buffer. Cells were lysed in 50 μl/well of lysis buffer. For measurement, 5 μl lysate of each well were transferred into a 384-well plate and proceeded as described before in detail.^96^

#### Alpha SureFire Ultra Multiplex pERK 1/2 & total ERK assay

The kit measures the phosphorylation and total levels of endogenous ERK1/2 in cellular lysates. The signal at 615 nm (Eu) corresponds to the pERK level, while the signal at 545 nm (Tb) corresponds to the total ERK level. The pERK/total ERK ratio of cell extracts was determined using the Alpha SureFire Ultra Multiplex p-ERK 1/2 (Thr202/Thr204)/ Total ERK assay according to the manufacturer (Revvity). CHO-K1 cells were split in 96-well plates 24 h after transfection and serum-starved overnight before the assay. 48 h after transfection, cells were stimulated with agonists in HBSS buffer (with 20 mM HEPES) for 15 min at 37 °C. Stimulation was stopped by aspiration of the media, and cells were lysed in 50 μl/well of the supplied lysis buffer with 250 μM protease inhibitor AEBSF. From each well, 10 μl of lysate were transferred into a 384-well plate, and acceptor beads and donor beads were added according to the manufacturer’s protocol. Measurements were taken using the EnVision Microplate reader (Revvity).

#### Bioluminescence resonance energy transfer (BRET) assay

24 h after transfection, HEK293-T cells were harvested and plated in white 96-well culture plates coated with poly-L-lysine. 16 h before the assay, cells were cultured in serum and niacin-free media. BRET assays were performed 72 h after transfection. Cells were washed with HBSS and incubated for 60 min in 32.5 μl HBSS/well at room temperature. 12.5 μl of Nluc substrate (Promega, diluted 1:250 in HBSS) were added, and three consecutive baseline reads were taken using the Victor Microplate reader (Revvity). Luminescence was measured at 460 ± 80 nm (acceptor) and 645 ± 75 nm (donor) for Nluc. BRET ratio was calculated as the ratio between acceptor and donor emission.^97^ Cells were stimulated by adding 5 μl of 10x ligand solution, and the measurement was continued for 20 min. The basal BRET ratio before ligand stimulation was defined as the average of three consecutive BRET values. To quantify ligand-induced changes, ΔBRET was calculated for each well as % over basal [(Ratio_stim_ - Ratio_basal_)/Ratio_basal_] x 100. Subsequently, the average ΔBRET of vehicle control was subtracted.^97^

#### Liquid chromatography-mass spectrometry (LC-MS) measurement

Fecal samples were collected non-invasively from the ground during routine animal husbandry procedures by the professional zoo keepers of the Zoo Leipzig and frozen at -20 °C. Before initial extraction, fecal samples were thawed, and 3-4 g were weighed in a 50 ml tube and filled up with 1xPBS to 30-40 ml, respectively (equals 0.1 g/ml). Samples were vortexed and shaken at 37 °C for 20 min. Subsequently, samples were centrifuged (5000 g) for 10 min. 12 ml of the supernatant was transferred to a 15 ml tube and again centrifuged (5000 g) for 10 min. Subsequently, the supernatant was sterile filtered (0.45 μM filter) and aliquoted (500 μl, 1:10 diluted fecal-PBS).

Sample preparation of 100 μl of these fecal extracts was performed as previously described.^98^ In detail, 900 μl of extraction buffer (90/10 v/v methanol: water), including internal standards (see supplementary), were added to the samples. The samples were shaken at 30 Hz for 2 min in a mixer mill, and proteins were precipitated at -20 °C for 2 h. The samples were centrifuged at 4 °C, 14,000 rpm (18620 g), for 10 min. 200 μL supernatant was transferred to microvials and evaporated to dryness in a speed-vac concentrator, and the samples were stored at -80 °C until analysis. Small aliquots of the remaining supernatants were pooled and used to create quality control (QC) samples.

Metabolic profiling by HILIC-LC-MS was performed at the Swedish Metabolomics Center in Umeå, Sweden. Solvents and eluent additives: Methanol and Acetonitrile, HPLC-grade, were obtained from Fischer Scientific (Waltham, MA, USA). Ammonium formate and Medronic acid (Methylenediphosphonic acid) were obtained from Sigma (St. Louis, MO, USA). Reference and tuning standards: Purine, HP-0921 (Hexakis(1H, 1H, 3H-tetrafluoropropoxy)phosphazine), 4 μM, 1 μM Calibrant, ESI-TOF, ESI-L Low Concentration Tuning Mix, HP-0321 (Hexamethoxyphosphazine), 0.1 mM were obtained from Agilent Technologies (Santa Clara, CA, USA). Stable isotope internal standards: ^13^C9-Phenylalanine, D_4_-Cholic acid, Salicylic acid-D_6_, L-glutamic acid-^13^C5,^15^N, and D-sucrose-^13^C12 were obtained from Sigma (St. Louis, MO, USA). L-proline-^13^C5 and alpha-ketoglutarate-^13^C4 were obtained from Cil (Andover, MA, USA).

Before the LC-MS analysis, the samples were re-suspended in 10 + 10 μl methanol and elution solvent A. The samples were analyzed according to a randomized run order. All samples were first analyzed in positive ionization mode, followed by a second injection in negative ionization mode.

The chromatographic separation was performed on an Agilent 1290 Infinity UHPLC system (Agilent Technologies, Waldbronn, Germany). 2 μL of each sample were injected onto an Atlantis Premier BEH-Z-HILIC VanGuard FIT (1.7 μm, 2.1 x 50 mm) column (Waters Corporation, Milford, MA, USA) held at 40 °C. The HILIC gradient elution solvents were A) H_2_O, 10 mM ammonium formate, 5μM Medronic acid, pH 9, B) 90:10 Acetonitrile: H_2_O, 10 mM ammonium formate, pH 9. Chromatographic separation was achieved using a linear gradient (at a flow rate of 0.4 ml/min) starting at 90 % B and decreasing to 80 % B over 6 min; B was reduced to 20 % over 3.5 min and held at 20 % for 1.5 min; B was increased to 90 % for 0.5 min and the flow-rate was increased to 0.7 ml/min for 2 min; these conditions were held for 0.5 min, after which the flow-rate was reduced to 0.4 ml/min for 0.5 min before the next injection.

The compounds were detected with an Agilent 6546 Q-TOF mass spectrometer equipped with a jet stream electrospray ion source operating in positive or negative ion mode. A reference interface was connected for accurate mass measurements; the reference ions purine (4 μM) and HP-0921 (Hexakis(1H, 1H, 3H-tetrafluoropropoxy)phosphazine) (1 μM) were infused directly into the MS at a flow rate of 0.05 ml/min for internal calibration, and the monitored ions were purine m/z 121.05 and m/z 119.03632; HP-0921 m/z 922.0098 and m/z 966.000725 for positive and negative mode respectively. The gas temperature was set to 150°C, the drying gas flow was set to 8 l/min, and the nebulizer pressure was 35 psi. The sheath gas temp was set to 350 °C, and the sheath gas flow was 11 l/min. The capillary voltage was set to 4000 V in both positive and negative ion mode. The nozzle voltage was 300 V. The fragmentor voltage was 120 V, the skimmer 65 V, and the OCT 1 RF Vpp 750 V. The collision energy was set to 0 V. The m/z range was 70 - 1700, and data was collected in centroid mode with an acquisition rate of 4 scans s^-1^. MS/MS analysis was run on the QC samples for identification purposes.

All data pre-processing was performed using the Agilent MassHunter Profinder version B.10.0.2 (Agilent Technologies Inc., Santa Clara, CA, USA). Data preprocessing was conducted in a targeted manner. A pre-defined list of metabolites commonly found in plasma and serum was searched for using the Batch Targeted feature extraction in MassHunter Profinder. An in-house HILIC-LC-MS library built from authentic standards and run on the same system with the same chromatographic and mass spectrometry settings was used for targeted processing.

### Quantification and statistical analysis

All experiments were performed at least three times, as indicated in the figure legends, and, unless otherwise indicated, they are presented as mean ± the standard error of the mean (SEM). EC_50_ values were determined in GraphPad Prism by fitting dose-dependence responses to a three-parameter sigmoidal curve (assuming a Hill slope of 1). Statistical analyses were performed using GraphPad Prism as indicated in the figure legends (Figures 1H, 3C, 3E, 4C, and 6B). P values with P ≤ 0.05 were considered statistically significant and are defined as follows:

∗P ≤ 0.05, ∗∗P≤ 0.01, ∗∗∗P ≤ 0.001.

## Supplemental information

Document S1. Figures S1–S6 and Tables S1, S3–S5, S8, and S9

Table S2. NCBI database accession numbers and sequence description of mammalian HCA_2_ orthologs, related to Figure 2

Table S6. Functional characterization of HCA_2_ orthologs stimulated with several agonists using cAMP inhibition assays, related to Figures 3, 5, and 6

Table S7. Conservation of orthosteric, extended, and allosteric ligand binding sites across 220 mammalian HCA_2_ orthologs, related to Figure 7
