# Supplementary material for: Comparative analysis reveals molecular adaptation of mammalian HCA2 to microbial metabolites
Source: iScience. 2026 May 22;29(6):116030. doi: 10.1016/j.isci.2026.116030 (PMC13217857; doi:10.1016/j.isci.2026.116030)
Supplement: Document S1. Figures S1–S6 and Tables S1, S3–S5, S8, and S9 [file mmc1.pdf]

iScience, Volume *29*

Supplemental information

Comparative analysis reveals molecular adaptation of mammalian HCA_2 to microbial metabolites

Franziska Bischof, Petra Krumbholz, Gunnar Kleinau, Patrick Scheerer, and Claudia Stäubert

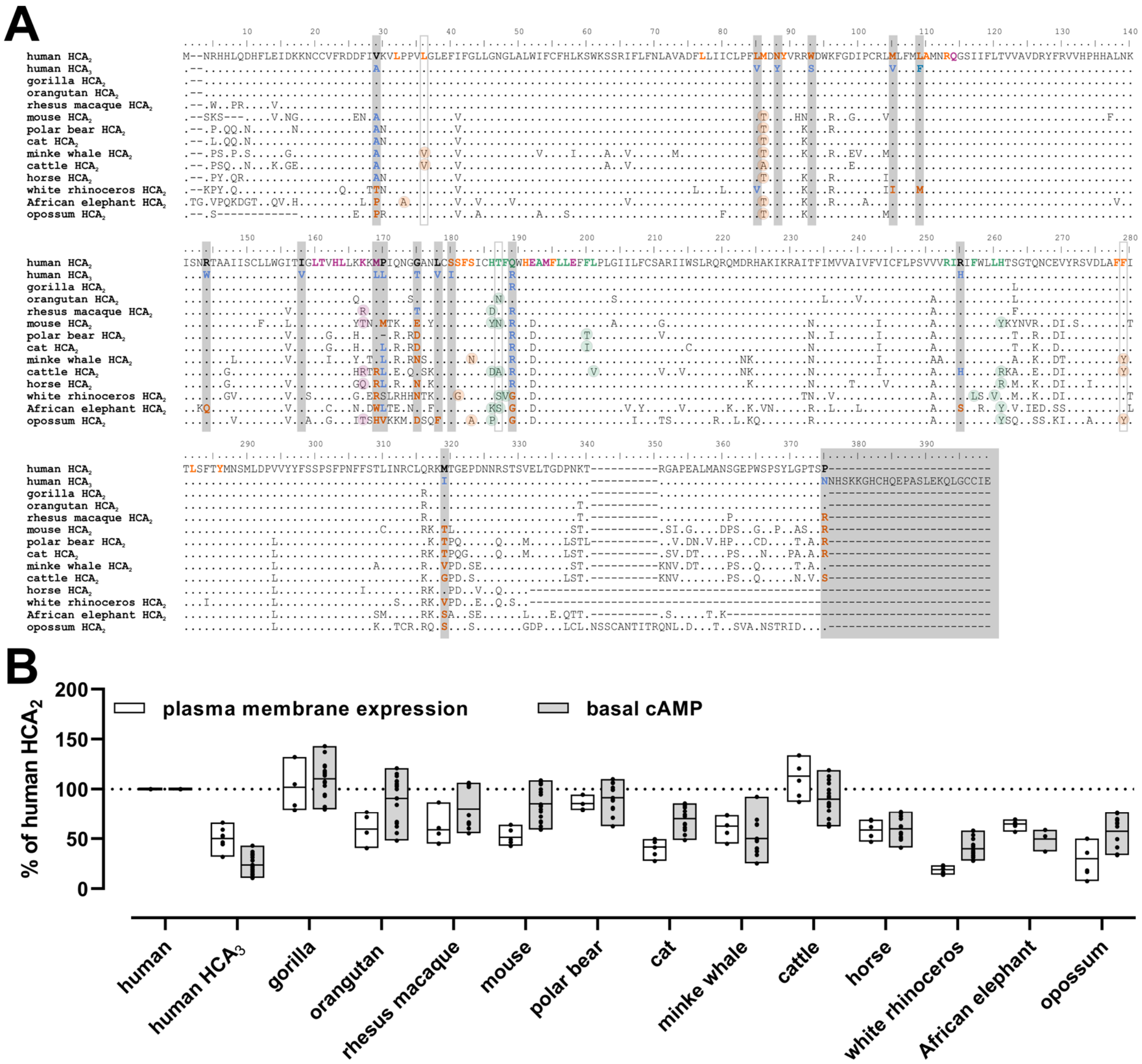

Figure S1: Protein alignment of cloned mammalian HCA_2 orthologs and human HCA_3, their plasma membrane expression, and basal activity. (A) Highlighted in gray are amino acid positions that differ between human HCA_2 and HCA_3. Blue letters: human HCA_3-specific amino acids. Red letters: amino acid positions differing from human HCA_2. **(B)** Cells were transiently transfected with HCA_2 orthologs or human HCA_3. Plasma membrane expression was determined using ELISA, and the basal inhibition of forskolin-induced cAMP accumulation was determined (shown as % of human HCA_2, data shown as min to max, line at mean, $n \geq 4$). Related to Figure 3.

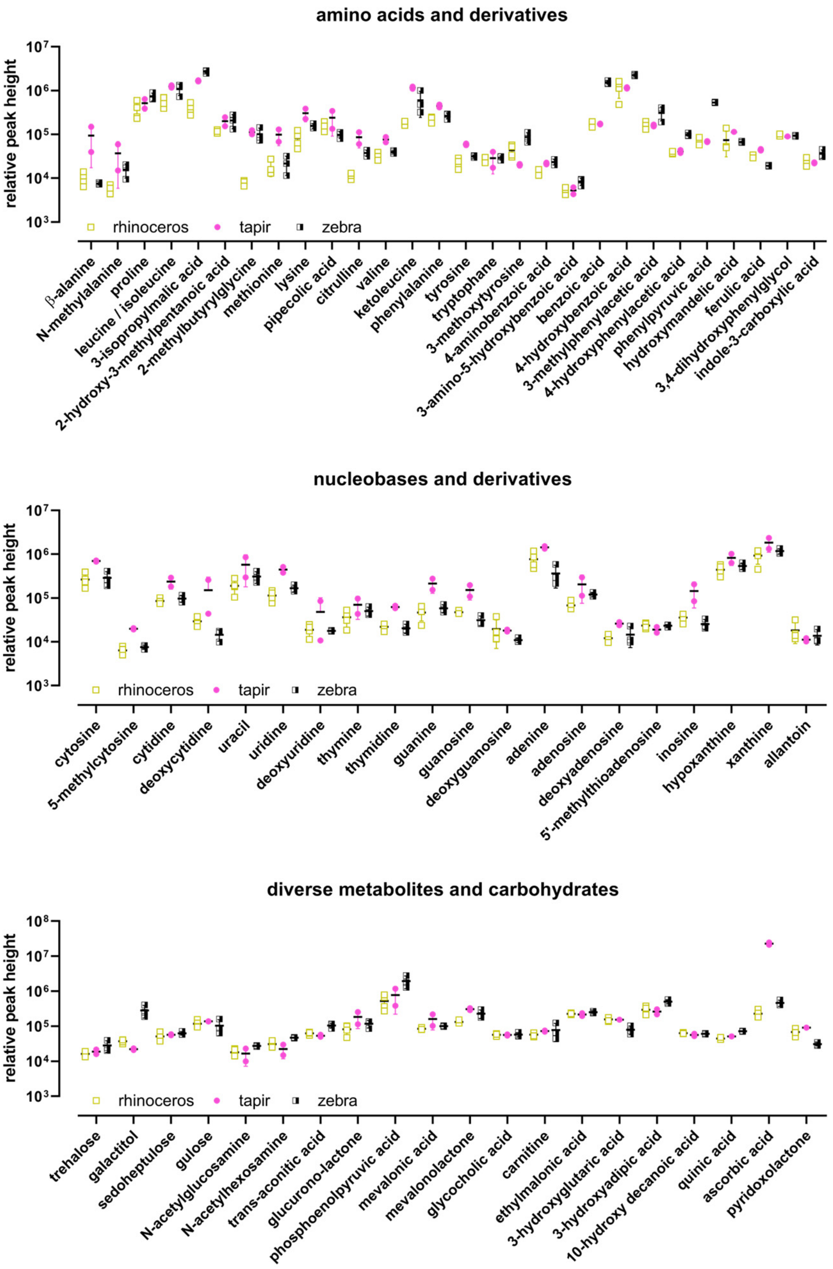

Figure S2: LC-MS-based metabolomics analyses of fecal sample extracts. Fecal samples were extracted, and hydrophilic interaction liquid chromatography (HILIC)-based metabolomics analyses were performed. Relative levels of detected and indicated metabolites are shown as relative peak height mean ± SD (black rhinoceros: n = 4, Asiatic tapir: n = 2, zebra: n = 3). Related to Figure 4.

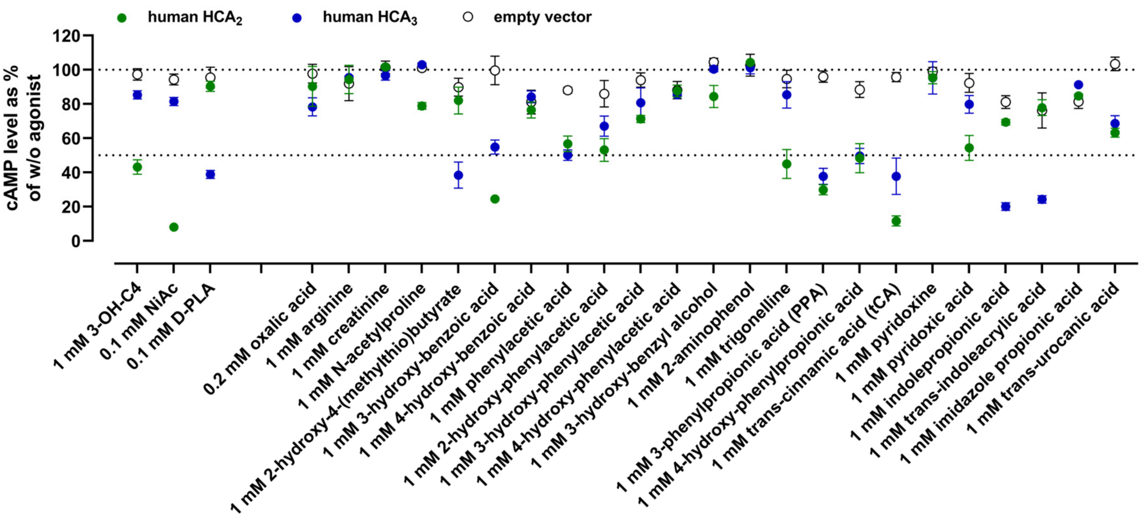

Figure S3: Metabolites identified in fecal samples of odd-toed ungulates activate human HCA_2 and HCA_3. Cells were transiently transfected with human HCA_2, HCA_3, or an empty vector control, and cAMP inhibition assays were performed in the presence of 2 µM forskolin upon stimulation with the indicated compounds (mean ± SEM, n = 3). Related to Figures 4, 5.

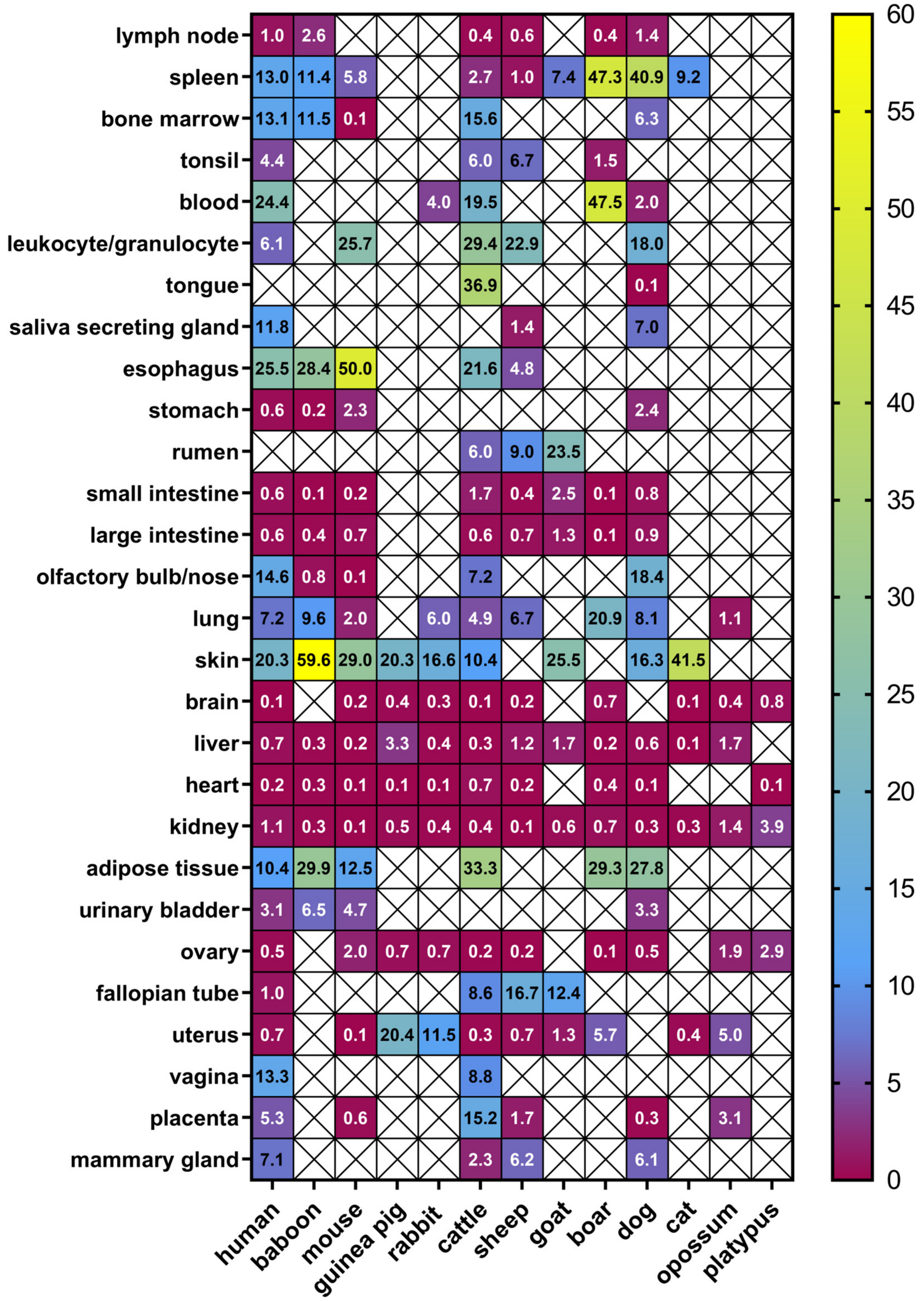

Figure S4: RNA expression data extracted from Bgee, a database of comparative transcriptomics in animals. TPM values were downloaded from https://www.bgee.org/ [1, 2] and are shown. X indicates that no data was available. Related to Figure 5

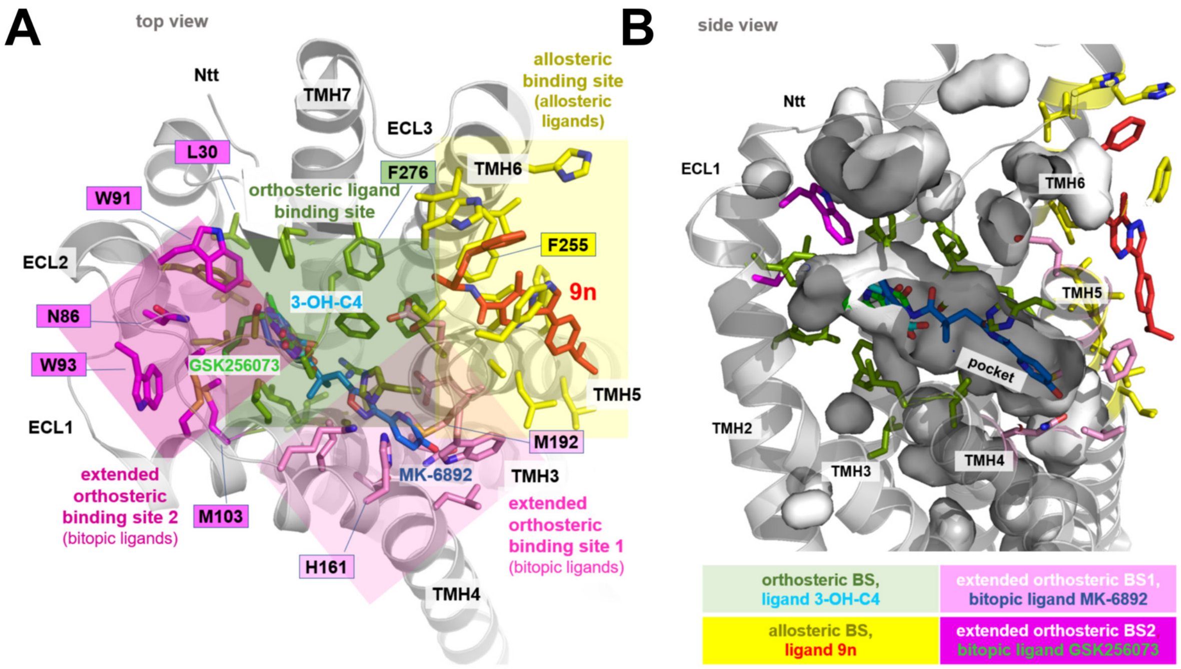

Figure S5: Ligand binding sites in determined HCA$_2$ complex structures. Several receptor-ligand complex structures in the protein data bank [3] (Table S4) represent active and inactive states, with or without G proteins, determined with diverse orthosteric, allosteric, or bitopic ligands. **(A)** Different ligands and surrounding amino acids (sticks) are superimposed and colour-coded to discriminate binding sites; only selected residues are labeled (full list in Table S9). The allosteric ligand 9n (yellow, PDB-ID 8j6q [4]) binds outside the orthosteric pocket (green, e.g. 3-OH-C4/NiAc). Several ligands engage residues beyond the orthosteric site, forming an "extended orthosteric binding site" (1 and 2)". These are defined as bitopic or dualsteric ligands [5], such as MK-6892 (PDB-ID 8ijd [6]), GSK256073 (PDB-ID 8k5d [6]), and acifran (PDB-ID 8ihi [7]). **(B)** A clipped "inner surface representation" reveals the ligand binding "pocket", here for MK-6892. Surrounding side chains either contact the ligand directly or form an extended binding shell, where substitutions may alter pocket shape and biophysical properties. All structural representations were generated using PyMol (Molecular Graphics System Version 2.5.5, Schrödinger, LLC, New York, NY). ECL: extracellular loop, TMH: transmembrane helix, Ntt: N-terminal tail. Related to Figure 7.

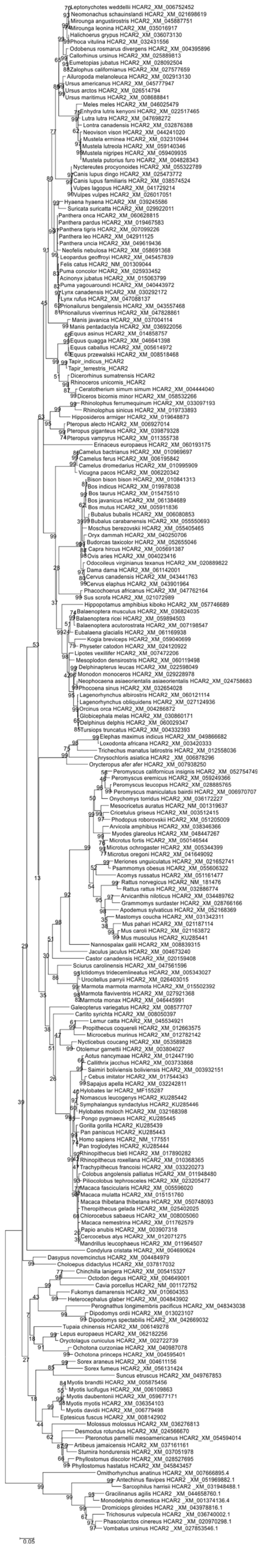

Figure S6: Bootstrap consensus phylogenetic tree of HCA_2 for 220 mammalian species. Molecular Phylogenetic analysis by Maximum Likelihood method.

The evolutionary history was inferred by using the Maximum Likelihood method based on the General Time Reversible model [8]. The tree with the highest log likelihood (-30866.9092) is shown. The percentage of trees in which the associated taxa clustered together is shown next to the branches. Initial tree(s) for the heuristic search were obtained automatically by applying Neighbor-Join and BioNJ algorithms to a matrix of pairwise distances estimated using the Maximum Composite Likelihood (MCL) approach, and then selecting the topology with superior log likelihood value. A discrete Gamma distribution was used to model evolutionary rate differences among sites (5 categories (+G, parameter = 0.6811)). The rate variation model allowed for some sites to be evolutionarily invariable ([+I], 30.8460% sites). The tree is drawn to scale, with branch lengths measured in the number of substitutions per site. The analysis involved 220 nucleotide sequences. Codon positions included were 1st+2nd+3rd+Noncoding. All positions with less than 95% site coverage were eliminated. That is, fewer than 5% alignment gaps, missing data, and ambiguous bases were allowed at any position. There were a total of 1023 positions in the final dataset. Evolutionary analyses were conducted in MEGA11 [9]. Related to STAR Methods.

Table S1

Ligand-dependent arrestin-3 recruitment of human HCA_2 using BRET. HEK-293T cells were co-transfected with HCA_2-mVenus and Nluc-tagged human arrestin-3. EC_{50} values were determined from concentration-response curves using GraphPad Prism. Data is given as mean ± SEM (EC_{50}) of at least three independent experiments, each performed in triplicate. Related to Figure 1.

	E_{max} (x-fold over vh)	EC_{50} (nM)
MK-6892	1.364 ± 0.047	77 ± 20
GSK256073	1.308 ± 0.038	230 ± 65
acifran	1.230 ± 0 035	17420 ± 6466
nicotinic acid	1.296 ± 0.039	493 ± 151
monomethylfumarate	1.255 ± 0.051	4077 ± 1126
acipimox	1.210 ± 0.035	17950 ± 10561

Table S3

Sequence conservation analyses of amino acid positions differing between human HCA_2 and HCA_3. HCA_2 sequence information from 220 mammalian species was aligned and analyzed for amino acid conservation at positions differing from human HCA_3. The numbers in brackets indicate the number of mammalian HCA_2 orthologs with the shown amino acid at this position. Related to Figure 2.

**Human HCA_2** amino acid position	**amino acid in Human HCA_2** (number of mammalian orthologs with this AA)	**amino acid in Human HCA_3** (number of mammalian orthologs with this AA)	**Amino acids occurring in other mammalian HCA_2 orthologs** (species with specified AA)
27 (1.32)	Val (22)	Ala (175)	Pro (12: *A. flavipes, D. gliroides, E. europaeus, E. maximus, G. agilis,* ***L. africana, M. domestica****, P. cinereus, S. etruscus, S. harrisii, T. Vulpecula, V. ursinus*)<br>Ser (5: *A. nancymaae, C. jacchus, C. imitator, S. boliviensis, S. apella*)<br>Thr (4: ***C. simum****, D. bicornis, D. sumatrensis, R. unicornis*)<br>Arg (2: *S. araneus, S. fumeus*)
83 (2.60)	Leu (217)	Val (3)	Val (3: ***C. simum****, D. bicornis, R. unicornis*)
86 (2.63)	Asn (220)	Tyr (0)	
91 (ECL2)	Trp (220)	Ser (0)	
103 (3.28)	Met (215)	Val (0)	Ile (3: *C. atys,* ***C. simum****, D. bicornis*)<br>Thr (2: *D. ordii, D. spectabilis*)
107 (3.32)	Leu (217)	Phe (0)	Met (3: ***C. simum****, D. bicornis, S. araneus*)
142 (4.40)	Arg (212)	Trp (0)	Gln (8: *E. maximus,* ***L. africana****, O. afer, M. javanica, M. pentadactyla, O. curzoniae, O. princeps, T. manatus*)
156 (4.54)	Ile (220)	Val (0)	
167 (ECL2)	Met (170)	Leu (6)	Arg (28: *B. bison, B. indicus, B. javanicus, B. mutus,* ***B. taurus****, B. bubalis, B. carabanensis, B. taxicolor, C. hircus, C. canadensis, C. elaphus, D. dama, M. berezovskii,* ***C. simum****, C. griseus, D. bicornis, R. unicornis, D. sumatrensis, E. asinus,* ***E. caballus****, E. przewalskii, E. quagga, O. virginianus, O. dammah, O. aries, T. indicus, T. terrestris, M. glareolus*)<br>Leu (6: *A. flavipes, L. catta, C. asiatica, O. degus, O. afer, S. harrisii*)<br>His (7: *D. gliroides, G. agilis,* ***M. domestica****, O. anatinus, P. cinereus, T. vulpecula, V. ursinus)*<br>Lys (3: *C. cristata, M. auratus, H. amphibius*)<br>Trp (2: *E. maximus,* ***L. africana***)<br>Ile (3: *M. javanica, M. pentadactyla, S. etruscus*)<br>Phe (1: *T. manatus*)
168 (ECL2)	Pro (18)	Leu (153)	Met (30: *A. russatus, A. sylvaticus, A. niloticus, A. amphibus, C. griseus, G. surdaster, M. coucha, M. unguiculatus, M. auratus, M. fortis, M. ochrogaster, M. oregoni, M. caroli,* ***M. musculus****, M. pahari, M. glareolus, N. galili, O. torridus, P. californicus, P. eremicus, P. leucopus, P. maniculatus, P. roborovskii, P. obesus, R. norvegicus, R. rattus, E. fuscus, R. ferrumequinum, R. sinicus, S. harrisii)*

			Val (8: *A. flavipes, D. gliroides, G. agilis, E. europaeus*, ***M. domestica***, *P. cinereus, T. vulpecula, V. ursinus*)<br>Gln (1: *C. porcellus*)<br>Ser (4: ***C. simum***, *D. bicornis, R. unicornis, H. lar*)<br>gap (6: *A. melanoleuca, H. hyaena, S. suricatta, U. americanus, U. arctos*, ***U. maritimus***)
173 (ECL2)	Gly (31)	Thr (15)	Asp (88)<br>Asn (46)<br>Thr (15: *C. atys, C. sabaeus, C. angolensis, M. fascicularis*, ***M. mulatta***, *M. nemestrina, M. thibetana, M. leucophaeus, P. anubis, R. bieti, R. roxellana, T. francoisi, T. gelada, I. tridecemlineatus, S. etruscus*)<br>Ser (11: *C. syrichta, C. lanigera, C. cristata, D. leucas, G. variegatus, H. glaber, O. degus, N. coucang, O. garnettii, P. tephrosceles, E. fuscus*)<br>Pro (10: *L. catta, M. murinus, P. coquereli, L. europaeus, F. damarensis, M. marmota, O. cuniculus, M. flaviventris, M. monax, S. carolinensis*)<br>Lys (5: *C. canadensis, C. porcellus, C. griseus, P. longimembris, N. galili*)<br>Ala (3: *O. curzoniae, O. princeps, T. chinensis*)<br>Glu (8: *A. sylvaticus, A. niloticus, G. surdaster, P. californicus, P. leucopus, M. coucha, M. caroli*, ***M. musculus***)<br>Arg (3: *D. ordii, D. spectabilis, M. monoceros*)
176 (ECL2)	Leu (210)	Val (0)	Phe (7: *D. gliroides, G. agilis*, ***M. domestica***, *O. anatinus, P. cinereus, T. vulpecula, V. ursinus*)<br>Arg (2: *A. flavipes, S. harrisii*)<br>Trp (1: *H. amphibious*)
178 (ECL2)	Ser (220)	Ile (0)	
187 (5.37)	Gln (5)	Arg (195)	Gly (19: *C. porcellus*, ***C. simum***, *C. asiatica, C. cristata, D. bicornis, R. unicornis, D. sumatrensis, A. flavipes, D. gliroides, E. maximus*, ***L. africana***, ***M. domestica***, *O. afer, P. cinereus, S. harrisii, T. manatus, T. Vulpecula, V. ursinus, H. amphibius*)<br>Gln (4: *C. lanigera*, ***H. sapiens***, ***P. pygmaeus,*** *F. damarensis, M. lucifugus*)<br>Trp (1: *P. tephrosceles*)<br>Leu (1: M. *berezovskii*)
253 (6.57)	Arg (194)	His (21)	His (21*: C. imitator, S. apella, B. bison, B. indicus, B. javanicus, B. mutus*, ***B. taurus***, *B. carabanensis, B. taxicolor, C. hircus, C. canadensis, C. elaphus, D. dama, M. berezovskii, O. virginianus, O. dammah, O. aries, P yagouaroundi, C. porcellus, M. javanica, M. pentadactyla*)<br>Ser (3: *E. maximus, T. manatus*, ***L. africana***)<br>Gly (1: *C. asiatica*)<br>Cys (1: *L. catta*)
317 (C term)	Met (31)	Ile (2)	Thr (77)<br>Val (27: ***C. simum***, *D. bicornis, D. sumatrensis, R. unicornis, T. indicus, T. terrestris*, ***B. acutorostrata***, *B. musculus, B. ricei, D.leucas,*

	D. delphis, E. glacialis, G. melas, K. breviceps, L. obliquidens, L. albirostris, L. vexillifer, M. densirostris, M. monoceros, N. asiaeorientalis, O. orca, P. sinus, P. catodon, T. truncatus, H. amphibious, P. africanus, S. scrofa)
	Ser (24: *L.europaeus, O. cuniculus, M. auratus, M. ochrogaster, M. fortis, M. oregoni, M. glareolus, A. amphibius, O. torridus, P. californicus, P. eremicus, P. leucopus, P. maniculatus, E.maximus,* ***L. africana****, T. manatus, C. asiatica, A. flavipes, D. gliroides, G. agilis,* ***M. domestica****, S. harrisii, V. ursinus*)
	Gly (16: *B. bison, B. indicus, B. javanicus, B. mutus,* ***B. taurus****, B. bubalis, B. carabanensis, B. taxicolor, C. hircus, C. canadensis, C. elpahus, D. dama, M. berezovskii, O. virginianus, O dammah, O. aries*)
	Ala (15: *E. lutris, L. canadensis, L. lutra, M. erminea, M. putorius, M. lutreola, M. nigripes, N. vison, O. curzoniae, O. princeps, M. molossus, P. discolor, P. hastatus, P. parnellii, T. chinensis*)
	Pro (15: *L.catta, M. murinus, N. coucang, O. garnettii, C. porcellus, C. lanigera, D. ordii, D. spectabilis, H. glaber, O. degus, P. longimembris, P. cinereus, O. anatinus, P. coquereli*)
	Lys (9: *E. fuscus, S. etruscus, M. lucifugus, M. davidii, M. brandtii, M. myotis, M. daubentonii, R. ferrumequinum, R. sinicus, D. rotundus*)
	Arg (3: *C. familiaris, C. dingo, E.europaeus*)
	Ile (2: *C. bactrianus, C. ferus*)
	Leu (1: *F. damarensis*)

Table S4

Collection of already published HCA2 and HCA3 receptor structures available via the PDB [3]. Related to Figure 2.

PDB code	Method	Resolution (Å)	Comment	Ligand (endogenous*, synthetic)	Ref.
8h2g	cryo-EM	3.01		niacin	10
8k5b	cryo-EM	3.43	without Gi	niacin	
8i7v	cryo-EM	2.77		acipimox	
8k5c	cryo-EM	3.13	without Gi	acipimox	
8i7w	cryo-EM	3.39		GSK256073	
8k5d	cryo-EM	3.74	without Gi	GSK256073	
8jz7	cryo-EM	2.60		MK-6892	11
8ijb	cryo-EM	3.28		acipimox	6
8ij3	cryo-EM	3.28		Apo-state	
8ijd	cryo-EM	3.25		MK-6892	
8ija	cryo-EM	2.69		niacin	
8j6q	cryo-EM	2.60	allosteric-orthosteric	9n-3HB (β-hydroxybutyrate)	4
8j6p	cryo-EM	2.55	allosteric-orthosteric	9n-niacin	
8j6r	cryo-EM	2.76		MK-6892	
8jhy	cryo-EM	2.87		9n (biased, allosteric)	12
8jii	cryo-EM	3.17		9n-niacin	
8jim	cryo-EM	2.98		MMF (monomethyl fumarate)	
8jil	cryo-EM	3.5		niacin	
7zl9	X-ray	2.70	inactive		13
7zly	X-ray	2.7	inactive		
7xk2	cryo-EM	3.1		MK-6892	
8ihb	cryo-EM	2.85		GSK256073	7
8ihf	cryo-EM	2.97		MK6892	
8ihh	cryo-EM	3.06		LUF6283	
8ihi	cryo-EM	3.11		acifran	
8ihj	cryo-EM	3.07		**HCAR3**-acifran-Gi	
8ihk	cryo-EM	3.07		**HCAR3**-acifran-Gi (local)	
8iy9	cryo-EM	3.37		niacin	14
8jer	cryo-EM	3.45		acipimox	
8iyh	cryo-EM	3.3		MK-6892	
8iyw	cryo-EM	3.45		GSK256073	
8jhn	cryo-EM	3.75		MMF	
8j6i	cryo-EM	2.92		MK-6892	15
8j6j	cryo-EM	2.8		GSK256073	
8j6l	cryo-EM	3.05		niacin	
9iqt	cryo-EM	2.9		niacin	16
8utd	cryo-EM	3.24		MK-1903	17
8uuj	cryo-EM	2.62		FCH-2296413 (DREADD)	
8jef	cryo-EM	2.96		3HO **(HCAR3)**	18
8jei	cryo-EM	2.73		5c **(HCAR3)**	

Table S5

Conservation of proposed G protein-interacting sites across 220 mammalian HCA_2 orthologs. The number in brackets indicates the number of species that have a certain amino acid at the specified position. Related to Figure 2.

G protein interaction sites	
Lys57^{ICL1} (220)	
Ser62$^{2.39}$ (220)	
Asp124$^{3.49}$ (220)	
Arg125$^{3.50}$ (220)	
Arg128$^{3.53}$ (220)	
Val129$^{3.54}$ (220)	
Pro132^{ICL2} (220)	
His133^{ICL2} (220)	
Lys138^{ICL2} (219)	Arg (1: *P. longimembris)*
Ile211$^{5.61}$ (220)	
Leu215^{ICL3} (220)	
Arg218^{ICL3} (220)	
Gln219^{ICL3} (217)	Lys (2: *L. africana, E. maximus*)<br>Asn (1: *P. catodon*)
Met220^{ICL3} (206)	Leu (14: *I. tridecemlineatus, M. flaviventris, M. marmota, M. monax, U. parryii, A. flavipes, D. gliroides, G. agilis, M. domestica, P. cinereus, S. harrisii, T. vulpecula, V. ursinus, O anatinus*)
Arg222^{ICL3} (171)	Lys (49)
His223^{ICL3} (207)	Gln (13: *C. syrichta, M. berezovskii, J. jaculus, C. didactylus, A. flavipes, D. gliroides, G. agilis, M. domestica, P. cinereus, S. harrisii, T. vulpecula, V. ursinus, O anatinusi)*
Lys225$^{6.29}$ (217)	Asn (2: *L. africana, E. maximus*)<br>Arg (1: *D. spectabilis*)
Ile226$^{6.30}$ (219)	Val (1: *D. spectabilis*)
Arg228$^{6.32}$ (218)	Lys (2: *R. ferrumequinum, R. sinicus*)
Ala229$^{6.33}$ (220)	
Phe232$^{6.36}$ (220)	
Ile233$^{6.37}$ (202)	Val (12: *P. africanus, S. scrofa, O. curzoniae, O. princeps, A. jamaicensis, D. rotundus, P. dicolor, P. hastatus, P. parnellii, P. cinereus, T. Vulpecula, V. ursinus*)<br>Leu (6: *P. pygmaeus, O. degus, S. fumeus, M. molossus, A. flavipes, S. harrisii*)
Ser297$^{7.56}$ (220)	
Ser298$^{8.47}$ (220)	
Pro299$^{8.48}$ (220)	

Table S8

Primers used for HCA_2 ortholog amplification, sequencing, and epitope-tag introduction. Related to STAR Methods.

ID	sequence (5´- 3´)	purpose
789	gtgcaaatcaaagaactgctcctc	pcDps forward (amplification/sequencing)
790	cctggttctttccgcctcagaag	pcDps reverse (amplification/sequencing)
2291	cgccgcactagttcacttatcgtcatcgtccttatagtc	FLAG-uni-*Spe I*_AS
2292	cgccgggtacctcactcacttatcgtcatcgtccttatagtc	FLAg-uni-*KpnI*_AS
2285	cgcgaattccccaccatgtaccccctacgacgtccccgactacgcc	HA-uni-Kozak-*Eco RI*_S
747	cctaggaccaactcgatcc	CsimumHCA2-S-5UTR
748	aacactcctggtagtactca	CsimumHCA2-AS-3UTR
749	ctccgtccaactaaccagat	Lafricana-HCA2-S-5UTR
750	gcttcttggctgggttaatt	LafricanaHCA2-AS-3UTR
751	ttccccgaaccttggcta	BtaurusHCA2-S-5UTR
752	gatcctggtgcttgatgatc	BtaurusHCA2-AS-3UTR
785	acgtccccgactacgccaccgggaacgtcc	LafricanaHCA2-HA-adaptor-S
786	acgtccccgactacgccaacccttcccaactg	BtaurusHCA2-HA-adaptor-S
787	acgtccccgactacgccaaaccgtaccaccag	CsimumHCA2-HA-adaptor-S
788	tcacttatcgtcatcgtccttatagtctttagccgttaaagcc	LafricanaHCA2-FLAG-adaptor-AS
789	tcacttatcgtcatcgtccttatagtcactggagactggattca	BtaurusHCA2-FLAG-adaptor-AS
790	tcacttatcgtcatcgtccttatagtcctctgtgtaccagctaaa	CsimumHCA2-FLAG-adaptor-AS
841	caacaccctgacatgacata	MmulattaHCA2-S-5UTR
842	cattacttgatgcaacagcc	MmulattaHCA2-AS-3UTR
843	ctaggaccagctcgatcc	EcaballusHCA2-S-5UTR
844	attaaagcgtctggaacact	EcaballusHCA2-AS-3UTR
845	tttcgtagcttcctggtaac	PtigrisHCA2-S-5UTR
846	attcctctgctgactttctg	PtigrisHCA2-AS-3UTR
847	ctcagacccacatcttgttc	BacutorostrataHCA2-S-5UTR
848	ccaaggctcatgatgttact	BacutorostrataHCA2-AS-3UTR
849	cctctttccttgagcactc	UmaritimusHCA2-S-5UTR
850	ccaagccaaactattttccc	UmaritimusHCA2-AS-3UTR
851	agccagttgtagagaggaaa	MdomesticaHCA2-S-5UTR
852	ttctcttttctctgccaaca	MdomesticaHCA2-AS-3UTR
859	acgtccccgactacgccaattggcaccatccg	MmulattaHCA2-HA-adaptor-S
860	tcacttatcgtcatcgtccttatagtcacgagaggttggacc	MmulattaHCA2-FLAG-adaptor-AS
861	acgtccccgactacgccaacccataccaccagc	EcaballusHCA2-HA-adaptor-S
862	tcacttatcgtcatcgtccttatagtcgacgcttgtgctctg	EcaballusHCA2-FLAG-adaptor-AS
863	acgtccccgactacgccaacctgcaccagca	PtigrisHCA2-HA-adaptor-S
875	tcacttatcgtcatcgtccttatagtcgcgagaagctgggg	PtigrisHCA2-FLAG-adaptor-AS
865	acgtccccgactacgccaacccgtcccacc	BacutorostrataHCA2-HA-adaptor-S
866	tcacttatcgtcatcgtccttatagtcaggagaggctggagt	BacutorostrataHCA2-FLAG-adaptor-AS
867	acgtccccgactacgccaacccgcaccagc	UmaritimusHCA2-HA-adaptor-S
868	tcacttatcgtcatcgtccttatagtcgttatttagagaagctggg	UmaritimusHCA2-FLAG-adaptor-AS
869	acgtccccgactacgccaacagcaaaaactgct	MdomesticaHCA2-HA-adaptor-S
870	tcacttatcgtcatcgtccttatagtcaggagacgttgggt	MdomesticaHCA2-FLAG-adaptor-AS
871	tggccatgaaccgc	mammalHCA2-300-S
872	tcacctacatgaacagca	mammalHCA2-800-S
1045	ttcaagcttgccaccatgaatcggcaccatctg	HindIII-Kozak-ATG-HCAR2-S
1046	tgcagaattcgaggagaggttgggcc	EcoRI-no-Stop-HCAR2-AS
1908	ggtgttgccgccggtggtggggctggagtttatc	huHCAR2_L34V-S
1909	gataaactccagccccaccaccggcggcaacacc	huHCAR2_L34V-AS
1924	gcttcagcatctgccatgccttccagtggcacgaag	huHCAR2_T185A-S
1925	cttcgtgccactggaaggcatggcagatgctgaagc	huHCAR2_T185A-AS
1926	cttcagcatctgccattccttccagtggcacg	huHCAR2_T185S-S
1927	cgtgccactggaaggaatggcagatgctgaag	huHCAR2_T185S-AS
1902	gtggacctggcgttctatatcactctcagcttc	huHCAR2_F277Y-S
1903	gaagctgagagtgatatagaacgccaggtccac	huHCAR2_F277Y-AS

Table S9

Sources of genomic DNA used for mammalian HCAR2 amplification. Related to STAR Methods.

**Species latin**	**Species english**	**Source**
Balaenoptera acutorostrata scammoni	Minke whale	Dr P. J. Palsboll, University of Wales, U.K.
Bos taurus	Cattle	Dr C. Pitra, IZW, Berlin, Germany
Ceratotherium simum	White rhinoceros	Dr C. Pitra, IZW, Berlin, Germany
Equus przewalskii	Horse	Dr C. Pitra, IZW, Berlin, Germany
Felis catus	Cat	Dr C. Pitra, IZW, Berlin, Germany
Loxodonta africana	African elephant	Dr C. Pitra, IZW, Berlin, Germany
Macaca mulatta	Rhesus monkey	Dr W. Enard, MPI, Leipzig, Germany
Monodelphis domestica	Gray short-tailed opossum	Dr C. Pitra, IZW, Berlin, Germany
Ursus maritimus	Polar bear	Dr C. Pitra, IZW, Berlin, Germany

References

1. Bastian, F.B., Cammarata, A.B., Carsanaro, S., Detering, H., Huang, W.-T., Joye, S., Niknejad, A., Nyamari, M., Mendes de Farias, T., and Moretti, S., et al. (2025). Bgee in 2024: focus on curated single-cell RNA-seq datasets, and query tools. Nucleic Acids Res *53,* D878-D885. 10.1093/nar/gkae1118.
2. Bastian, F.B., Roux, J., Niknejad, A., Comte, A., Fonseca Costa, S.S., Farias, T.M. de, Moretti, S., Parmentier, G., Laval, V.R. de, and Rosikiewicz, M., et al. (2021). The Bgee suite: integrated curated expression atlas and comparative transcriptomics in animals. Nucleic Acids Res *49,* D831-D847. 10.1093/nar/gkaa793.
3. Berman, H.M., Westbrook, J., Feng, Z., Gilliland, G., Bhat, T.N., Weissig, H., Shindyalov, I.N., and Bourne, P.E. (2000). The Protein Data Bank. Nucleic Acids Res *28,* 235–242. 10.1093/nar/28.1.235.
4. Mao, C., Gao, M., Zang, S.-K., Zhu, Y., Shen, D.-D., Chen, L.-N., Yang, L., Wang, Z., Zhang, H., and Wang, W.-W., et al. (2023). Orthosteric and allosteric modulation of human HCAR2 signaling complex. Nat Commun *14,* 7620. 10.1038/s41467-023-43537-z.
5. Mohr, K., Schmitz, J., Schrage, R., Tränkle, C., and Holzgrabe, U. (2013). Molecular alliance-from orthosteric and allosteric ligands to dualsteric/bitopic agonists at G protein coupled receptors. Angewandte Chemie (International ed. in English) *52,* 508–516. 10.1002/anie.201205315.
6. Pan, X., Ye, F., Ning, P., Zhang, Z., Li, X., Zhang, B., Wang, Q., Chen, G., Gao, W., and Qiu, C., et al. (2023). Structural insights into ligand recognition and selectivity of the human hydroxycarboxylic acid receptor HCAR2. Cell Discov *9,* 118. 10.1038/s41421-023-00610-7.
7. Suzuki, S., Tanaka, K., Nishikawa, K., Suzuki, H., Oshima, A., and Fujiyoshi, Y. (2023). Structural basis of hydroxycarboxylic acid receptor signaling mechanisms through ligand binding. Nature communications *14,* 5899. 10.1038/s41467-023-41650-7.
8. Nei, M., and Kumar, S. (2000). Molecular evolution and phylogenetics (Oxford University Press).
9. Tamura, K., Stecher, G., and Kumar, S. (2021). MEGA11: Molecular Evolutionary Genetics Analysis Version 11. Molecular biology and evolution *38,* 3022–3027. 10.1093/molbev/msab120.
10. Park, J.-H., Kawakami, K., Ishimoto, N., Ikuta, T., Ohki, M., Ekimoto, T., Ikeguchi, M., Lee, D.-S., Lee, Y.-H., and Tame, J.R.H., et al. (2023). Structural basis for ligand recognition and signaling of hydroxy-carboxylic acid receptor 2. Nature communications *14,* 7150. 10.1038/s41467-023-42764-8.
11. Cheng, L., Sun, S., Wang, H., Zhao, C., Tian, X., Liu, Y., Fu, P., Shao, Z., Chai, R., and Yan, W. (2023). Orthosteric ligand selectivity and allosteric probe dependence at Hydroxycarboxylic acid receptor HCAR2. Signal transduction and targeted therapy *8,* 364. 10.1038/s41392-023-01625-y.
12. Zhao, C., Wang, H., Liu, Y., Cheng, L., Wang, B., Tian, X., Fu, H., Wu, C., Li, Z., and Shen, C., et al. (2023). Biased allosteric activation of ketone body receptor HCAR2 suppresses inflammation. Molecular cell *83,* 3171-3187.e7. 10.1016/j.molcel.2023.07.030.
13. Yang, Y., Kang, H.J., Gao, R., Wang, J., Han, G.W., DiBerto, J.F., Wu, L., Tong, J., Qu, L., and Wu, Y., et al. (2023). Structural insights into the human niacin receptor HCA2-Gi signalling complex. Nature communications *14,* 1692. 10.1038/s41467-023-37177-6.
14. Yadav, M.K., Sarma, P., Maharana, J., Ganguly, M., Mishra, S., Zaidi, N., Dalal, A., Singh, V., Saha, S., and Mahajan, G., et al. (2024). Structure-guided engineering of biased-agonism in the human niacin receptor via single amino acid substitution. Nat Commun *15,* 1939. 10.1038/s41467-024-46239-2.
15. Zhu, S., Yuan, Q., Li, X., He, X., Shen, S., Wang, D., Li, J., Cheng, X., Duan, X., and Xu, H.E., et al. (2023). Molecular recognition of niacin and lipid-lowering drugs by the human hydroxycarboxylic acid receptor 2. Cell reports *42,* 113406. 10.1016/j.celrep.2023.113406.
16. Liu, Y., Zhou, Z., Guan, F., Han, Z., Zhu, C., Ye, S., Yu, X., and Qiao, A. (2024). Ligand Recognition and Activation Mechanism of the Alicarboxylic Acid Receptors. Journal of molecular biology *436,* 168795. 10.1016/j.jmb.2024.168795.
17. Kang, H.J., Krumm, B.E., Tassou, A., Geron, M., DiBerto, J.F., Kapolka, N.J., Gumpper, R.H., Sakamoto, K., Dewran Kocak, D., and Olsen, R.H.J., et al. (2024). Structure-guided design of a peripherally restricted chemogenetic system. Cell *187,* 7433-7449.e20. 10.1016/j.cell.2024.11.001.
18. Ye, F., Pan, X., Zhang, Z., Xiang, X., Li, X., Zhang, B., Ning, P., Liu, A., Wang, Q., and Gong, K., et al. (2024). Structural basis for ligand recognition of the human hydroxycarboxylic acid receptor HCAR3. Cell reports *43,* 114895. 10.1016/j.celrep.2024.114895.
